# Supervised learning-guided design and CNC fabrication of Schwarz TPMS with experimental evaluation for atmospheric water generation

**DOI:** 10.1038/s41598-026-46613-8

**Published:** 2026-05-25

**Authors:** Md Shafikul Islam, Bahram Asiabanpour

**Affiliations:** Ingram School of Engineering, Texas State University, San Marcos, TX 78666 USA

**Keywords:** Atmospheric water generation (AWG), Triply periodic minimal surface (TPMS), Supervised learning, Subtractive manufacturing, Thermoelectric cooling (TEC), Engineering, Materials science

## Abstract

Atmospheric Water Generation (AWG) offers a decentralized and sustainable solution to global water scarcity by extracting moisture from the air. This study enhances AWG performance by combining supervised learning–guideddesign optimization and experimental validation of Schwarz Triply Periodic Minimal Surface (TPMS) structures. TPMS geometries improve condensation through a combination of large surface area and enhanced thermal conduction, enabling efficient droplet shedding. Structural and thermal simulations in nTop software generated data for predicting the top surface temperature—critical for condensation—based on parameters such as lattice thickness, cell size, surface area, and solid volume. Among the supervised learning models tested (Support Vector Machine, K-Nearest Neighbor, and Decision Tree), the Decision Tree showed the best accuracy (R² = 0.9676), captuirng both linear and nonlinear relationships. Using optimized parameters, a Schwarz TPMS structure was fabricated from Aluminum 6061-T6 via multi-axis CNC milling through a layer-wise subtractive method. Experimental evaluation under thermoelectric cooling (4–7 V) showed that the TPMS outperformed a solid aluminum block at 5 V and 6 V, achieving up to 33% higher water yield, though performance declined at 7 V due to frost formation. Thermal mapping highlighted non-uniform cooling from interfacial resistance between layers. This work demonstrates a scalable approach that integrates supervised learning–guided design with subtractive manufacturing for efficient AWG systems.

## Introduction

### Global water scarcity and atmospheric water generation (AWG)

Water scarcity has emerged as a critical global issue, impacting millions across various regions. Although freshwater makes up only about 2.5% of the Earth’s total water resources, its availability for safe human consumption is rapidly declining due to factors such as climate change, population growth, and pollution^1^. Since the early 20th century, global water usage has surged—particularly in Asia and worldwide^2^. Key freshwater sources, including rivers, lakes, and aquifers, are increasingly overused or polluted, often rendering them unsuitable for drinking. In addition, arid and semi-arid regions face heightened difficulties due to low rainfall and frequent droughts^3^. These escalating challenges highlight the urgent need for innovative and sustainable solutions, especially in areas where conventional water supply methods are no longer effective.

AWG is gaining recognition as a promising solution to freshwater shortages by utilizing an abundant yet often overlooked resource—moisture in the air^4^. AWG systems work by extracting and condensing water vapor from the atmosphere, providing a sustainable and decentralized method of producing potable water even in regions facing extreme environmental challenges^5^. Despite its potential, the widespread implementation of AWG faces barriers such as high energy demands, elevated production costs, and inconsistent performance under varying climate conditions.

On the technological front, research efforts have concentrated on improving both condensation efficiency and energy usage in AWG systems. Traditional AWG setups typically rely on vapor compression refrigeration (VCR) to cool air below its dew point, triggering condensation. However, newer approaches, such as thermoacoustic refrigeration (TAR), which generates temperature gradients using sound waves, have been investigated for their energy-efficient and environmentally friendly potential^6^. In addition, bioinspired AWG designs that replicate water-harvesting strategies observed in nature—like the microstructures on desert beetles or plant leaves—have shown encouraging results in enhancing condensation performance^7^. Furthermore, the adoption of hybrid hydrophilic-superhydrophobic surfaces has advanced fog collection techniques, providing a complementary passive mechanism that can enhance AWG systems^8^.

### Surface engineering and TPMS structures for condensation

In AWG systems, the design and treatment of the condensation surface are critical for improving water yield, as they directly influence droplet formation, movement, and heat transfer performance^9^. Research has consistently shown that enhancing surface area through macro-, micro-, and nano-scale structuring can significantly boost condensation rates. For example, superhydrophobic surfaces engineered with micro- and nanopillars have been found to reduce droplet size variation and increase the heat transfer coefficient by up to 26.4%, thereby enabling more effective water collection^10^. Additionally, lubricant-infused nano-textured surfaces enhance droplet mobility and minimize droplet pinning, allowing for continuous removal of condensed droplets and improved condensation efficiency^11^.

Surface roughness at the nanoscale also plays a vital role, as it can confine droplet bases and encourage spherical droplet formation, promoting better coalescence and movement^12^. Importantly, dropwise condensation offers a heat transfer rate that is more than ten times higher than that of film-wise condensation, making it a preferred mode for efficient thermal management^13^.

TPMS structures are mathematically defined geometries known for their high surface-area-to-volume ratios, making them highly effective in enhancing condensation processes in AWG systems^14^. Common TPMS forms—such as Gyroid, Schwarz, Diamond, Lidinoid, Split-P, and Neovius—are described by specific mathematical equations and are readily available as design templates in software tools like nTop^15^. These structures can be fabricated using both subtractive techniques, such as multi-axis CNC machining, and additive manufacturing methods like 3D printing, offering flexibility in design and production^16^. Their integration into AWG systems can lead to improved condensation efficiency on the cold side of thermoelectric modules, reduce re-evaporation of collected moisture, and potentially lower manufacturing costs^17^.

### Machine learning (ML) for AWG optimization

Recent advances in machine learning (ML) provide powerful tools for optimizing complex engineering systems, including AWG systems^18^. Conventional AWG surface design methods primarily rely on empirical testing, trial-and-error experimentation, and limited parametric studies, which become increasingly time-consuming, costly, and inefficient when multiple geometric and thermal variables are involved^19^.

In contrast, supervised learning models can serve as efficient surrogate predictors by learning underlying relationships between geometric design parameters and thermal performance directly from simulation and experimental data. These data-driven models enable rapid evaluation of large design spaces, facilitate multi-parameter optimization, and significantly reduce the need for repeated physical prototyping^20^.

### Interplay between material, geometry, and manufacturing process

In the fabrication of complex structures, the relationship between material choice, manufacturing method, and geometric design is essential for optimizing performance and efficiency^21^. The selected material affects key attributes such as mechanical strength, machinability, and thermal behavior, all of which are critical in applications like AWG. The manufacturing process, whether subtractive or additive, governs how the material can be shaped, with each approach offering different advantages and limitations. Geometry—especially in the context of TPMS—is instrumental in enhancing surface-area-to-volume ratios and boosting condensation performance^22,23^.

However, producing intricate TPMS geometries using subtractive manufacturing poses significant challenges due to the complexity of tool paths, accessibility issues, and limitations in material removal. The successful realization of such structures depends on the careful balance and integration of these three elements: suitable material selection, process capabilities, and geometric intricacy. This synergy ultimately determines the feasibility and effectiveness of the fabrication approach.

### Subtractive manufacturing as a scalable alternative for TPMS fabrication

TPMS geometries, characterized by their intricate, interconnected forms and high surface-area-to-volume ratios, have primarily been produced using Additive Manufacturing (AM) methods such as laser deposition, material extrusion, and powder bed fusion. Although AM is well-suited for creating complex structures—especially for prototyping or low-volume production—it has several notable drawbacks. Metal-based AM processes are typically time-consuming and costly, often reaching hundreds of dollars per part, and may result in residual stresses, variable mechanical properties, and rough surface finishes. These issues pose significant barriers to large-scale implementation where cost-efficiency, material consistency, and production speed are critical^16,24,25^.

Multi-axis CNC machines allow for simultaneous movement along multiple axes, including rotational motion, enabling precise fabrication of complex geometries with better tool access. This capability makes them particularly effective for machining TPMS designs that include intricate internal features and complex contours. In contrast to conventional 3-axis systems that require multiple setups, 5-axis CNC machines can reach various angles in a single operation, reducing both errors and machining time. Beyond geometric flexibility, CNC machining provides more scalable and cost-effective than AM for mass production^26,27^.

### Research gap and objectives

Although significant progress has been made in TEC-based AWG systems, most existing research has concentrated on optimizing operational parameters such as voltage, airflow, and ambient humidity, while continuing to rely on conventional flat or finned aluminum cooling surfaces. For example, Milani et al.^28^ demonstrated that increasing the surface area of the cooler can enhance condensation efficiency, particularly in hot and humid environments, and Liu et al.^29^ emphasized the role of air contact time and surface geometry in water production. However, these studies remained limited to planar or simple surface designs, leaving the potential of advanced geometries underexplored.

Subsequent research has focused on material and system-level improvements rather than structural redesigns of the condensation surface. For instance, He et al.^30^ used hydrophobic coatings to promote dropwise condensation, and Kadhim et al.^31^ introduced solar-powered TEC-AWG systems to reduce energy dependence. Similarly, Abdul-Wahab et al.^32^ investigated multi-stage TEC configurations to boost water yield. While valuable, these efforts did not consider geometry as a performance-enhancing parameter, thereby overlooking how surface design itself can drive efficiency gains.

Meanwhile, bioinspired and textured surfaces have demonstrated the importance of geometry in condensation processes. Park et al.^33^ explored beetle-inspired structures for passive dew collection, and Chen et al.^34^ examined patterned copper surfaces for improved droplet shedding. Yet, such approaches have been largely confined to passive dew or fog harvesting systems, with minimal crossover into active TEC-driven AWG setups.

The integration of TPMS into TEC-AWG systems remains almost entirely unexplored. TPMS geometries offer inherently high surface-area-to-volume ratios, interconnected porosity for enhanced thermal conduction, and favorable droplet-shedding characteristics. Numerous studies have investigated TPMS structures primarily from mechanical and structural performance perspective. For example, Qiu et al.^35^ investigated the mechanical behavior and energy absorption capacity of several TPMS lattices using experimental compression testing and finite element modeling, demonstrating their superior crashworthiness and deformation characteristics. Similarly, Wang et al.^36^ examined composite TPMS porous structures inspired by bone microstructures and reported enhanced energy absorption and crashworthiness compared with conventional foam materials. In another study, Sadeghi et al.^37^ analyzed the thermomechanical behavior of TPMS metamaterials fabricated from shape memory polymers, highlighting their tunable stiffness and structural performance across different volume fractions. Despite these promising attributes, little is known about how their geometric parameters influence condensation and thermal behavior in AWG applications. Existing literature has not examined the interplay between TPMS geometry and surface temperature, nor has it systematically distinguished between linear and non-linear effects of geometric variables. A notable exception is the conceptual work of Tang et al.^38^, which proposed minimal surfaces for heat exchanger optimization but did not extend this concept to AWG. This lack of experimental validation and predictive modeling underscores a critical gap in the field and highlights the need for studies that link advanced surface geometries with thermal performance in TEC-based AWG systems.

Building on our recently published work on multi-axis CNC machining of TPMS geometries, which demonstrated that Schwarz and Neovius structures can be fabricated cost-effectively in Aluminum 6061-T6, the manufacturability of TPMS surfaces for AWG applications has now been established^16^. However, that earlier study focused solely on fabrication feasibility, toolpath strategies, and cost analysis, without examining how TPMS geometries actually perform during thermoelectric cooling. As a result, the functional relationship between TPMS geometric parameters and AWG performance—specifically their effects on surface temperature, thermal conduction pathways, frost formation behavior, and overall condensation efficiency—remains unknown.

Sharma et al.^39^ have demonstrated the effectiveness of supervised machine learning for predicting mechanical properties of polymer/carbon nanotube composites fabricated through microwave-assisted processing. In their work, Random Forest, Support Vector Regression, and K-Nearest Neighbors models were successfully applied to predict hardness and tensile strength under both ambient and elevated-temperature conditions, achieving high predictive accuracy. In parallel, Yan et al.^40^ proposed a multi-objective ML-driven framework for optimizing stiffness, strength, and energy absorption of TPMS structures, while Hussain et al.^41^ developed an automated ML-based recommendation system for TPMS parameters in material extrusion fabrication. Similarly, Mishra^42^ integrated machine learning with simulated annealing to optimize tensile stress in TPMS architectures, and Abbas et al.^43^ employed artificial neural networks to guide the design of multi-material TPMS lattices for enhanced mechanical behavior.

Despite these advances, existing ML-driven TPMS studies have predominantly focused on mechanical strength, stiffness, and energy absorption under structural loading, with limited attention to thermal transport, condensation dynamics, or phase-change behavior. Moreover, most reported frameworks rely on additive manufacturing routes and simulation-based mechanical validation, with minimal consideration of subtractive manufacturing constraints, thermoelectric cooling conditions, or interfacial thermal resistance effects. This gap motivates the present study, which extends machine learning–based optimization to TPMS-enabled condensation surfaces for TEC-based AWG conditions.

The objective of this study is to develop and validate a design methodology that improves AWG performance through the following specific aims:

- To generate structural and thermal simulation data using Schwarz TPMS geometries, analyzing parameters such as lattice thickness, cell size, surface area, and solid volume, in order to predict the top surface temperature critical for condensation.
- To train and compare supervised learning models such as SVM, KNN and Decision Tree using the simulated data, identify the model with the highest predictive accuracy, and understand how linear and nonlinear relationships among geometry and thermal parameters affect condensation.
- To fabricate optimized Schwarz TPMS structures in Aluminum 6061-T6 via multi-axis CNC milling (layer-wise subtractive manufacturing) based on the model’s design recommendations.
- To experimentally evaluate the AWG performance of the optimized TPMS structures under thermoelectric cooling (4–7 V), compare their performance against a solid aluminum block, and investigate factors (e.g., frost formation, interfacial resistance) that may reduce effectiveness at higher voltages.

Through achieving these aims, this work intends to deliver a scalable, machine learning–guided design + fabrication framework for enhancing AWG systems.

## Materials and methods

**Fig. 1:**
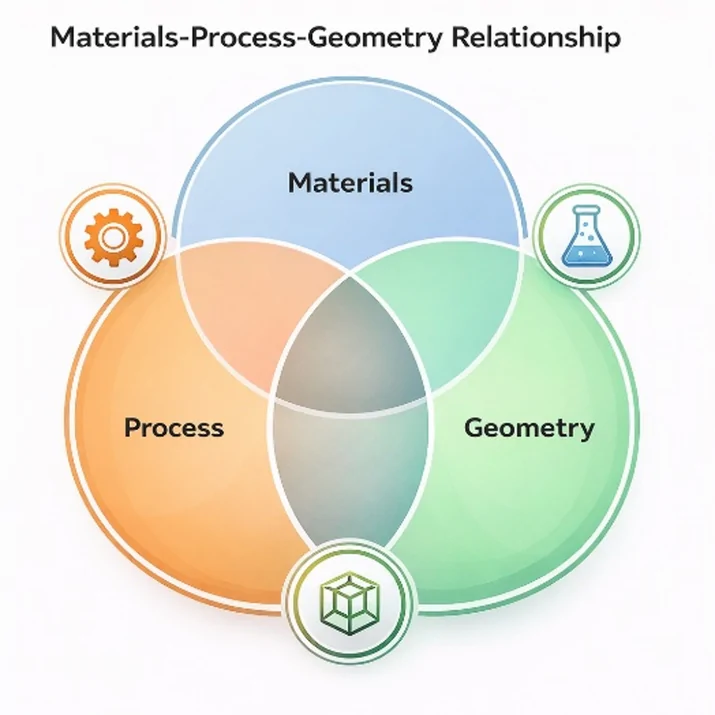
Venn diagram illustrates the intersection of materials, process, and geometry.

Figure 1 illustrates the critical interplay among materials, process, and geometry—a foundational relationship in advanced manufacturing. In this study, the selected material is Aluminum 6061-T6, chosen for its excellent thermal conductivity, corrosion resistance, and machinability, all of which are essential for enhancing condensation in AWG systems^44^. The geometry employed is the Schwarz, known for its high surface-area-to-volume ratio and interconnected porous structure, making it ideal for promoting efficient condensation and heat transfer^45^. The manufacturing process is multi-axis CNC milling, which offers the precision and flexibility needed to fabricate complex geometries like TPMS with high dimensional accuracy and superior surface finish^36^. The successful integration of these three elements—material, process, and geometry—defines the viability of fabricating functional, scalable components for next-generation water harvesting systems.

### Material

Aluminum 6061-T6 is selected as the material of choice for fabricating AWG components due to its excellent balance of thermal performance, machinability, durability, and cost-effectiveness. With a thermal conductivity ranging from approximately 150–230 W/m·K, it enables efficient and uniform heat transfer, which is essential for condensing atmospheric moisture in AWG systems^46^. This makes it especially suitable for thermal components like cooling plates and heat exchangers. Compared to high-performance metals such as copper or titanium, aluminum 6061 offers significant cost advantages in both raw material sourcing and machining, while its lightweight nature further reduces transportation and handling costs^47,48^. Known for its excellent machinability, the alloy supports precise and efficient manufacturing of complex geometries—such as TPMS structures—with reduced tool wear and shorter production cycles. Moreover, its inherent corrosion resistance due to a natural oxide layer ensures long-term durability in the high-humidity environments typical of AWG applications.

### Geometry

**Fig. 2:**
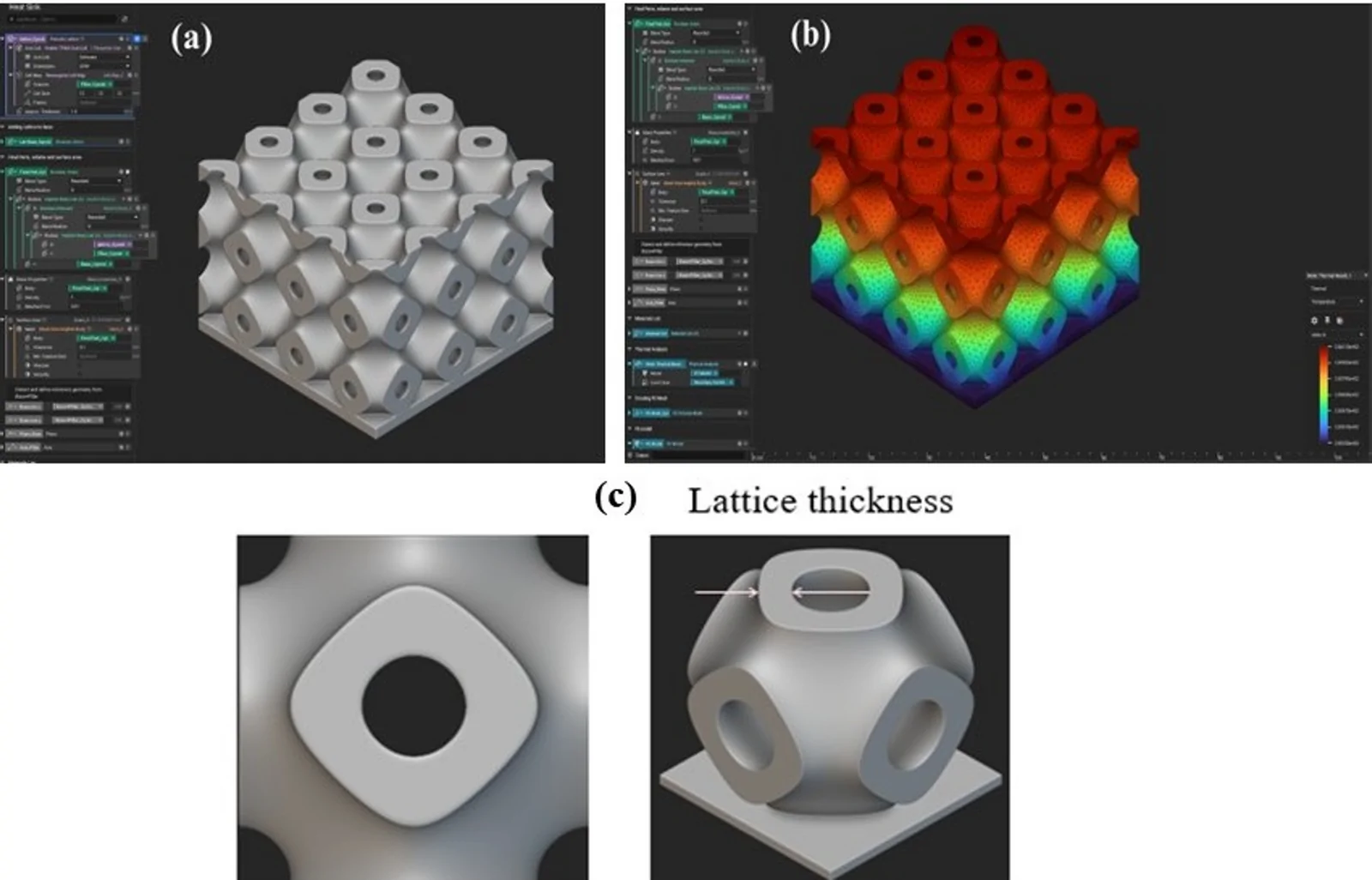
(**a**) Schwarz TPMS geometry modeled in nTop (**b**) Thermal simulation showing uniform heat distribution across the structure (**c**) Single Schwarz TPMS cell showing the top view (left) and isometric view with indicated lattice thickness (right).

TPMS structures, particularly Schwarz geometry, offer unique advantages that make them highly suitable for enhancing condensation in AWG systems. As shown in Fig. 2(a), these geometries consist of a continuous and periodic porous network that maximizes the surface-area-to-volume ratio while maintaining mechanical stability. This extensive surface area promotes greater droplet nucleation, while the interconnected voids facilitate improved airflow and efficient thermal dissipation, both of which are critical to the performance of TEC-based AWG systems^49^.

Among the various TPMS topologies available, the Schwarz geometry was selected in this study because it provides a favorable balance between thermal performance and manufacturability. Previous investigations on CNC-based fabrication of TPMS structures have shown that several geometries, such as Gyroid, Diamond, and Lidinoid, encounter significant tool accessibility and collision limitations during subtractive machining, whereas the Schwarz structure can be fabricated with comparatively higher geometric fidelity and machining stability make^16^. In addition to its manufacturability advantages, the Schwarz geometry maintains a well-distributed pore network and continuous solid pathways, which are beneficial for both heat conduction and condensation processes in thermoelectric AWG systems.

From a mathematical perspective, TPMS structures are defined by periodic minimal surface equations that generate smooth, continuous surfaces with zero mean curvature. Such mathematical regularity enables precise control over geometric parameters, including lattice thickness, unit cell size, and porosity, which directly influence thermal conduction pathways and exposed condensation area.

The high surface-area-to-volume ratio (SA/V) of Schwarz TPMS structures plays a central role in improving condensation performance and can be expressed as Eq. 1.

1 $$\:\mathrm{S}\mathrm{A}/\mathrm{V}=\frac{\mathrm{A}}{\mathrm{V}}$$

where $\:\mathrm{A}$ represents the total exposed surface area and $\:\mathrm{V}$ denotes the volume of solid material. Higher SA/V values increase the available nucleation sites for droplet formation while maintaining sufficient conductive pathways for heat transfer.

In addition to enhancing surface availability, the interconnected solid network provides continuous thermal conduction paths between the TEC cold side and the exposed condensation surface. According to Fourier’s law of heat conduction, the heat transfer rate through the TPMS structure is given Eq. 2.

2 $$\:\mathrm{q}=-\mathrm{k}\mathrm{A}\frac{\mathrm{d}\mathrm{T}}{\mathrm{d}\mathrm{x}}$$

where $\:\mathrm{q}$ is the heat flux, $\:\mathrm{k}$ is the thermal conductivity of aluminum, $\:\mathrm{A}$ is the effective conduction area, and $\:\frac{\mathrm{d}\mathrm{T}}{\mathrm{d}\mathrm{x}}$ represents the temperature gradient. By distributing conductive pathways throughout the porous network, Schwarz TPMS geometries promote more uniform cooling across the surface.

Furthermore, the curved and periodic nature of the Schwarz surface facilitates efficient droplet transport and shedding. The characteristic curvature reduces contact line pinning and encourages droplet coalescence and gravitational drainage, thereby limiting surface flooding and re-evaporation. The droplet nucleation density ($\:{\mathrm{N}}_{\mathrm{d}}$) on such structured surfaces is influenced by both surface roughness and available area and may be approximated as Eq. 3.

3 $$\:{\mathrm{N}}_{\mathrm{d}}\propto\:{\mathrm{A}}_{\mathrm{eff}}$$

where $\:{\mathrm{A}}_{\mathrm{eff}}$ denotes the effective wetted surface area available for condensation.

#### Modeling in nTop

The Schwarz TPMS geometry was designed using nTop, a parametric modeling software well-suited for generating complex 3D structures. nTop provides flexible control over key design parameters such as lattice thickness, cell size, and porosity, allowing for precise tuning of the geometry to meet functional requirements. The overall geometry was modeled with dimensions of 40 mm × 40 mm × 30 mm, where the 40 mm × 40 mm footprint was specifically chosen to match the size of the Peltier module’s cold surface, ensuring optimal thermal contact during condensation experiments. Additionally, nTop supports thermal simulations, enabling designers to evaluate whether the geometry promotes uniform heat distribution—a crucial factor in maximizing condensation efficiency. Figure 2(a) displays the Schwarz geometry modeled in nTop, highlighting its interconnected structure and periodic surface pattern. Figure 2(b) shows the uniform temperature distribution from cold to hot.

High thermal conductivity is essential for efficient heat transfer during condensation, while a high surface area increases the potential for water collection. Additionally, designing geometry to improve machinability helps reduce fabrication time, cost, and material waste. A larger lattice thickness also contributes to better thermal conduction by providing more continuous solid paths for heat flow. Figure 2(c) illustrates a single Schwarz TPMS cell, highlighting the structural features and the defined lattice thickness.

#### Data generation

Repetitive data were taken using nTOP for supervised learning, where cell size and lattice thickness are selected as the primary input factors for data generation. Through structural simulations, variations in these input parameters facilitate the calculation of the surface area of the geometry and the volume of the solid. These two properties are integral to determining the performance of the geometries in thermal and condensation-related applications. Using the surface area and volume values, the surface area-to-volume ratio is calculated, which is a critical factor influencing heat transfer efficiency. Additionally, the cell density is computed as the ratio of the volume of the solid to the volume of the block. The block volume is kept constant at 40 mm × 40 mm × 30 mm, representing the chosen dimensions of the geometry to maintain consistency across simulations. However, it is important to note that these simulations are based on ideal conditions and do not account for potential real-world factors such as surface roughness, manufacturing defects, or environmental variability.

The simulations begin with the smallest feasible parameters: a cell size of 4 mm × 4 mm × 4 mm and a lattice thickness of 0.5 mm. Incremental increases are systematically applied, with the cell size and lattice thickness progressing stepwise. The cell size increases from 4 mm × 4 mm × 4 mm to 5 mm × 5 mm × 5 mm, 6 mm × 6 mm × 6 mm, and so on, until reaching a maximum of 25 mm × 25 mm × 25 mm. Simultaneously, the lattice thickness increases in steps of 0.125 mm, ranging from 0.5 mm to a maximum of 2.5 mm. This systematic variation of parameters is intended to generate a comprehensive dataset with 236 data points, covering a wide range of geometric configurations.

**Fig. 3:**
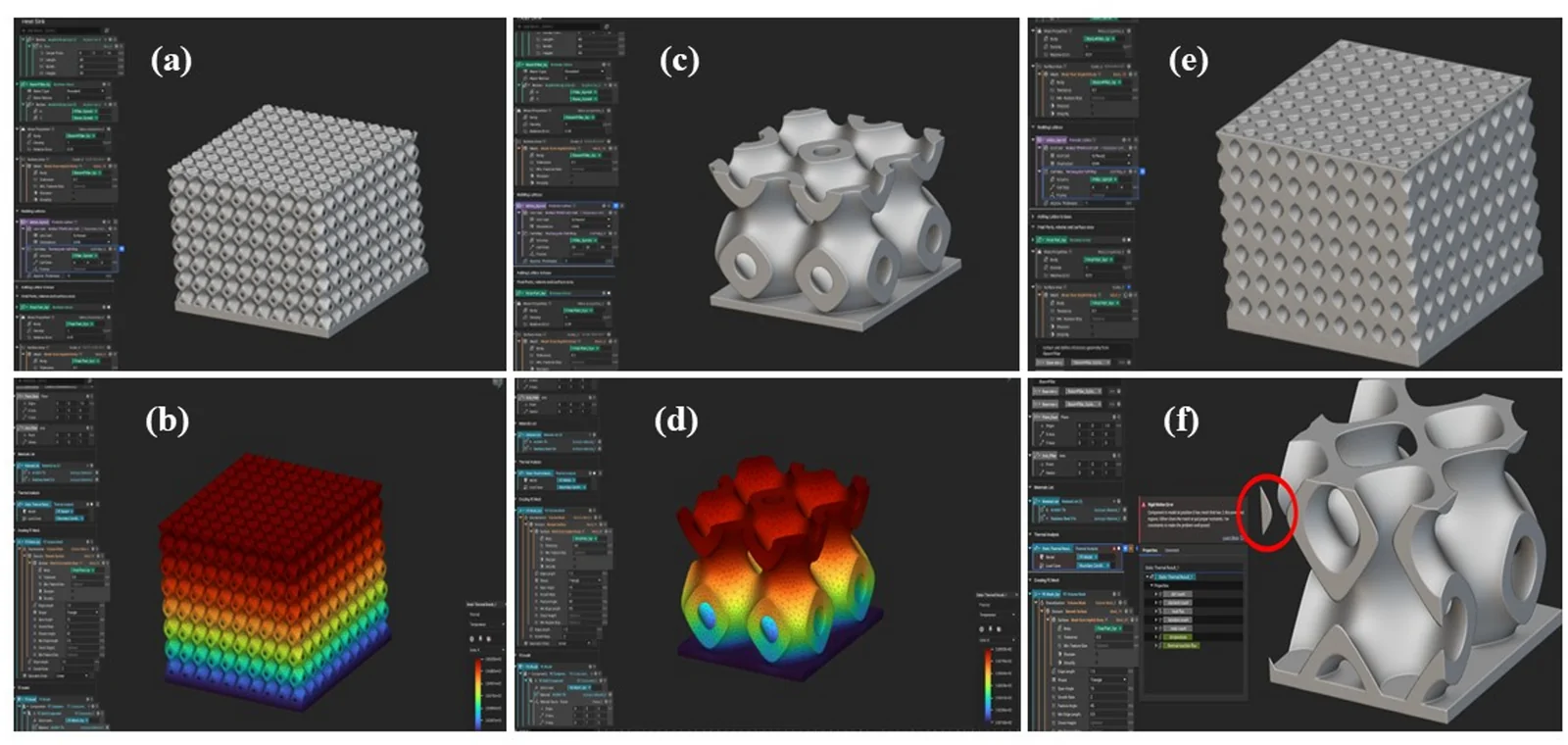
Structural and thermal simulations of Schwarz structure for varying cell sizes and lattice thicknesses: (**a**,** b**) Schwarz structure with 4 mm × 4 mm × 4 mm cell size and 0.5 mm lattice thickness; (**c**,** d**) Schwarz structure with 20 mm × 20 mm × 20 mm cell size and 2 mm lattice thickness; (**e**) Undeveloped Schwarz structure with 4 mm × 4 mm × 4 mm cell size and 1 mm lattice thickness, (**f**) Broken Schwarz structure with 24 mm × 24 mm × 24 mm cell size and 1.25 mm lattice thickness.

Figure 3 illustrates Schwarz structures generated with varying cell sizes and lattice thicknesses. A cell size below 4 mm × 4 mm produces a solid block, while sizes above 25 mm × 25 mm × 25 mm create overly hollow geometries unsuitable for AWG. Similarly, lattice thickness below 0.5 mm leads to weak, unstable structures, whereas thickness above 2.5 mm reduces porosity and exposed surface area. These limitations ultimately decrease condensation efficiency.

However, during structural simulations, certain cell size and lattice thickness combinations fail to generate valid Schwarz geometries, resulting in solid blocks or broken/incomplete structures. These invalid configurations are omitted from the final dataset to ensure relevance and accuracy. Figure 3(e) and (f) illustrate examples of excluded data, highlighting structures that failed to meet the required geometric criteria for further analysis.

To clarify the scope of these simulations, the simulations conducted in nTop primarily capture the geometric and conductive thermal characteristics of the TPMS structures rather than the full condensation process. The framework incorporates the thermal conductivity of Aluminum 6061-T6 and applies steady-state thermal conditions to estimate surface temperature distributions across different geometries. Because condensation in AWG systems occurs when the surface temperature drops below the ambient dew point, the predicted top surface temperature was used as a practical indicator of condensation potential. The physical relevance of the simulation-guided design was subsequently examined through experimental thermoelectric cooling tests, as presented in Sect.  3.4.

### Data collection and supervised learning

After excluding invalid combinations, a total of 236 data points was finalized for the Schwarz geometry. These configurations met the structural criteria and were analyzed for parameters such as surface area, solid volume, cell density, and other features relevant to thermal performance. Feature preprocessing included converting descriptive unit cell dimensions (e.g., “4 × 4 × 4”) into numerical edge lengths and applying one-hot encoding to the categorical variable “Cell Type (CT)”. This refined dataset provides a consistent and reliable basis for modeling the relationship between geometric factors and top surface temperature.

Although the dataset contains a moderate number of samples, machine learning models were adopted because the relationship between TPMS geometric parameters and thermal behavior is inherently nonlinear and multi-variable. Parameters such as lattice thickness, cell size, surface area, and solid volume interact in complex ways that are difficult to represent using predefined functional forms typically assumed in classical regression or response surface models. In contrast, supervised learning algorithms can learn nonlinear relationships directly from data without requiring explicit assumptions about the governing mathematical structure. This capability makes machine learning particularly suitable for capturing the complex geometric–thermal interactions present in TPMS-based condensation surfaces.

To predict the top surface temperature, three supervised learning models—SVM, KNN, and Decision Tree regression—were performed in Python using the Scikit-learn library, with Pandas and NumPy employed for numerical handling and Matplotlib for visualization. The dataset was randomly partitioned into training and testing subsets using an 80/20 split with a fixed random seed (random_state = 42) to ensure reproducibility. The supervised regression problem can be expressed as Eq. 4.

4 $$\:{\mathrm{T}}_{\mathrm{p}\mathrm{r}\mathrm{e}\mathrm{d}}=\mathrm{f}\left(\mathbf{X}\right)$$

where $\:{\mathrm{T}}_{\mathrm{p}\mathrm{r}\mathrm{e}\mathrm{d}}$ is the predicted top surface temperature and X = [TL, CS, SA/V, CD, A_s_, V_s_] represents the input feature vector consisting of lattice thickness (TL), cell size (CS), surface-area-to-volume ratio, cell density, surface area, and solid volume.

The SVM model employed a radial basis function (RBF) kernel defined as Eq. 5

5 $$\:\mathrm{K}({\mathbf{x}}_{\mathrm{i}},{\mathbf{x}}_{\mathrm{j}})=\mathrm{e}\mathrm{x}\mathrm{p}\left((-{\upgamma\:}\parallel\:{\mathbf{x}}_{\mathrm{i}}-{\mathbf{x}}_{\mathrm{j}}{\parallel\:}^{2})\right)$$

which enables nonlinear mapping of input features into higher-dimensional space. Default hyperparameters were used in this study.

For the KNN model, predictions were computed as the average of the k nearest neighbors, expressed in Eq. 6.

6 $$\:{\mathrm{T}}_{\mathrm{p}\mathrm{r}\mathrm{e}\mathrm{d}}=\frac{1}{\mathrm{k}}\sum_{\mathrm{i}=1}^{\mathrm{k}}{\mathrm{T}}_{\mathrm{i}}$$

where $\:{\mathrm{T}}_{\mathrm{i}}$ are the temperature values of the k closest samples identified using Euclidean distance. In this work, $\:\mathrm{k}=5$.

The Decision Tree model partitions the feature space into hierarchical regions by minimizing intra-node variance, producing a piecewise constant prediction function. The regressor was implemented with default splitting criteria and a fixed random seed (random_state = 42).

Model performance was assessed on the held-out test dataset using standard regression metrics defined as Eqs. 7–10.

7 $$\:\mathrm{M}\mathrm{S}\mathrm{E}=\frac{1}{\mathrm{n}}\sum\:_{\mathrm{i}=1}^{\mathrm{n}}({\mathrm{T}}_{\mathrm{i}}-{\mathrm{T}}_{\mathrm{p}\mathrm{r}\mathrm{e}\mathrm{d},\mathrm{i}}{)}^{2}$$

8 $$\:\mathrm{M}\mathrm{A}\mathrm{E}=\frac{1}{\mathrm{n}}\sum\:_{\mathrm{i}=1}^{\mathrm{n}}\mid\:{\mathrm{T}}_{\mathrm{i}}-{\mathrm{T}}_{\mathrm{p}\mathrm{r}\mathrm{e}\mathrm{d},\mathrm{i}}\mid\:$$

9 $$\:\mathrm{R}\mathrm{M}\mathrm{S}\mathrm{E}=\sqrt{\mathrm{M}\mathrm{S}\mathrm{E}}$$

10 $$\:{\mathrm{R}}^{2}=1-\frac{\sum\:_{\mathrm{i}=1}^{\mathrm{n}}({\mathrm{T}}_{\mathrm{i}}-{\mathrm{T}}_{\mathrm{p}\mathrm{r}\mathrm{e}\mathrm{d},\mathrm{i}}{)}^{2}}{\sum\:_{\mathrm{i}=1}^{\mathrm{n}}({\mathrm{T}}_{\mathrm{i}}-\stackrel{\prime }{\mathrm{T}}{)}^{2}}$$

These metrics provided a rigorous and reproducible basis for comparing predictive accuracy and evaluating the reliability of the developed models for thermal behavior prediction in TEC-based AWG systems.

All datasets and machine learning scripts used in this study are publicly available in a Zenodo repository (DOI: https://doi.org/10.5281/zenodo.19212706) to ensure reproducibility of the results.

### Process

#### Toolpath generation in mastercam

After modeling, the geometry was exported as a STEP file and imported into Mastercam for toolpath generation. Due to the intricate nature of the Schwarz geometry and the compact block dimensions of 40 mm × 40 mm × 30 mm, fabricating the entire structure in a single run using subtractive manufacturing was not feasible. To overcome this limitation, it was decided to fabricate the geometry in mirrored half-layers, with each half being machined separately and then stacked together to reconstruct the full structure. This approach proved to be more practical, simplifying the machining process and reducing tool accessibility challenges.

**Fig. 4:**
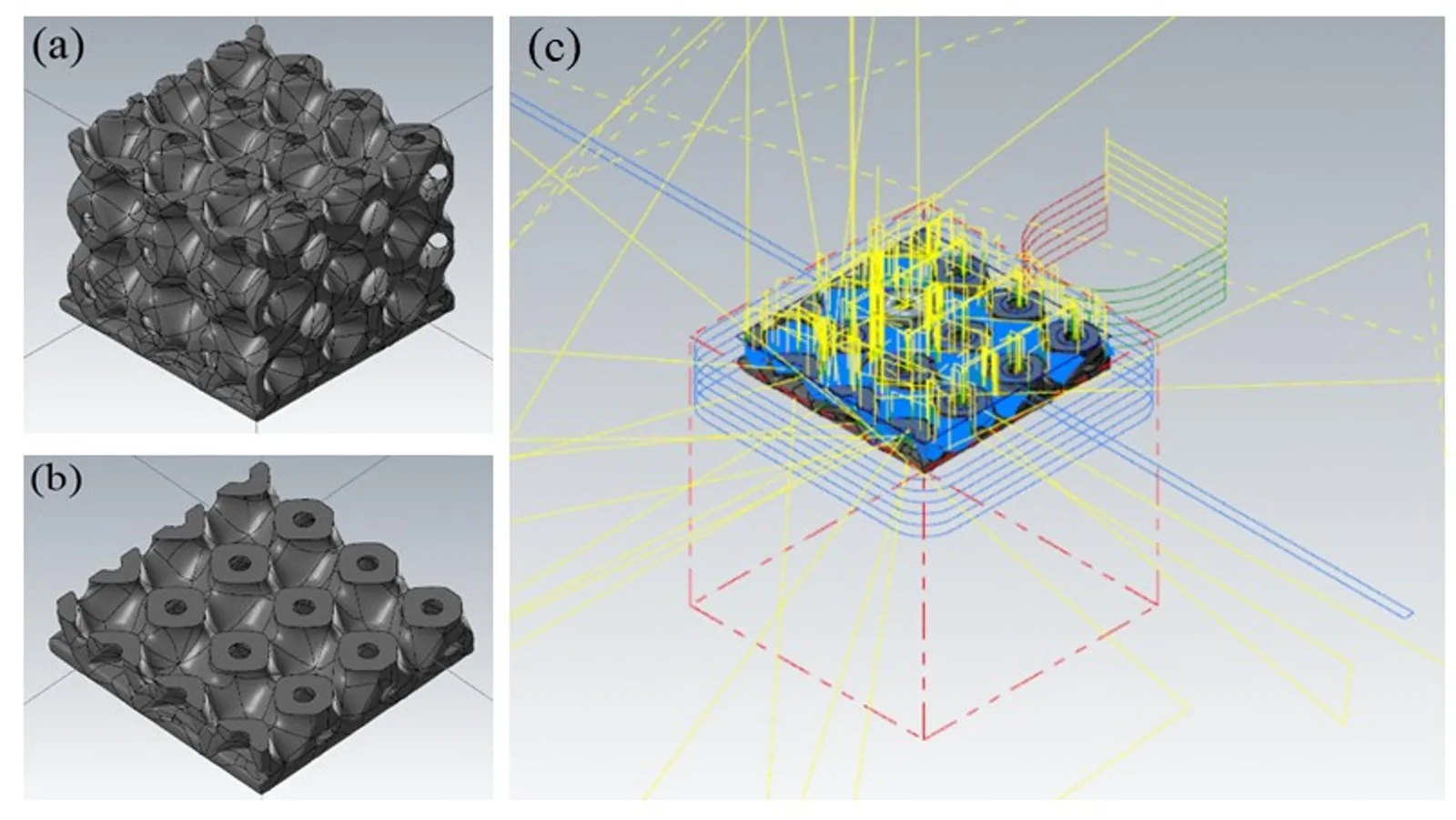
(**a**) Full Schwarz block, (**b**) Extracted top layer of Schwarz using Boolean cut, (c) Toolpath generation for Schwarz top single layer.

To machine the Schwarz geometry, a top-layer slice was extracted from the full 40 mm × 40 mm × 30 mm block model, as illustrated in Fig. 4(b). The complete Schwarz structure prior to slicing is shown in Fig. 4(a). A detailed wireframe model was generated on the extracted top layer to assist in precisely machining the uneven, pocket-like features of the surface, as shown in Fig. 4(c). The toolpath strategy began with facing using a ½-inch flat end mill to level the surface, followed by contouring to define outer boundaries. For the intricate inner contours and smoother surface transitions, surface finish flowline operations were executed using a 1/16-inch end mill. Finally, a 1/32-inch flat end mill was employed to machine fine pocket details, ensuring fidelity to the complex geometry while minimizing tool deflection and preserving edge sharpness.

#### Simulation in mastercam and machining of schwarz single layer

**Fig. 5:**
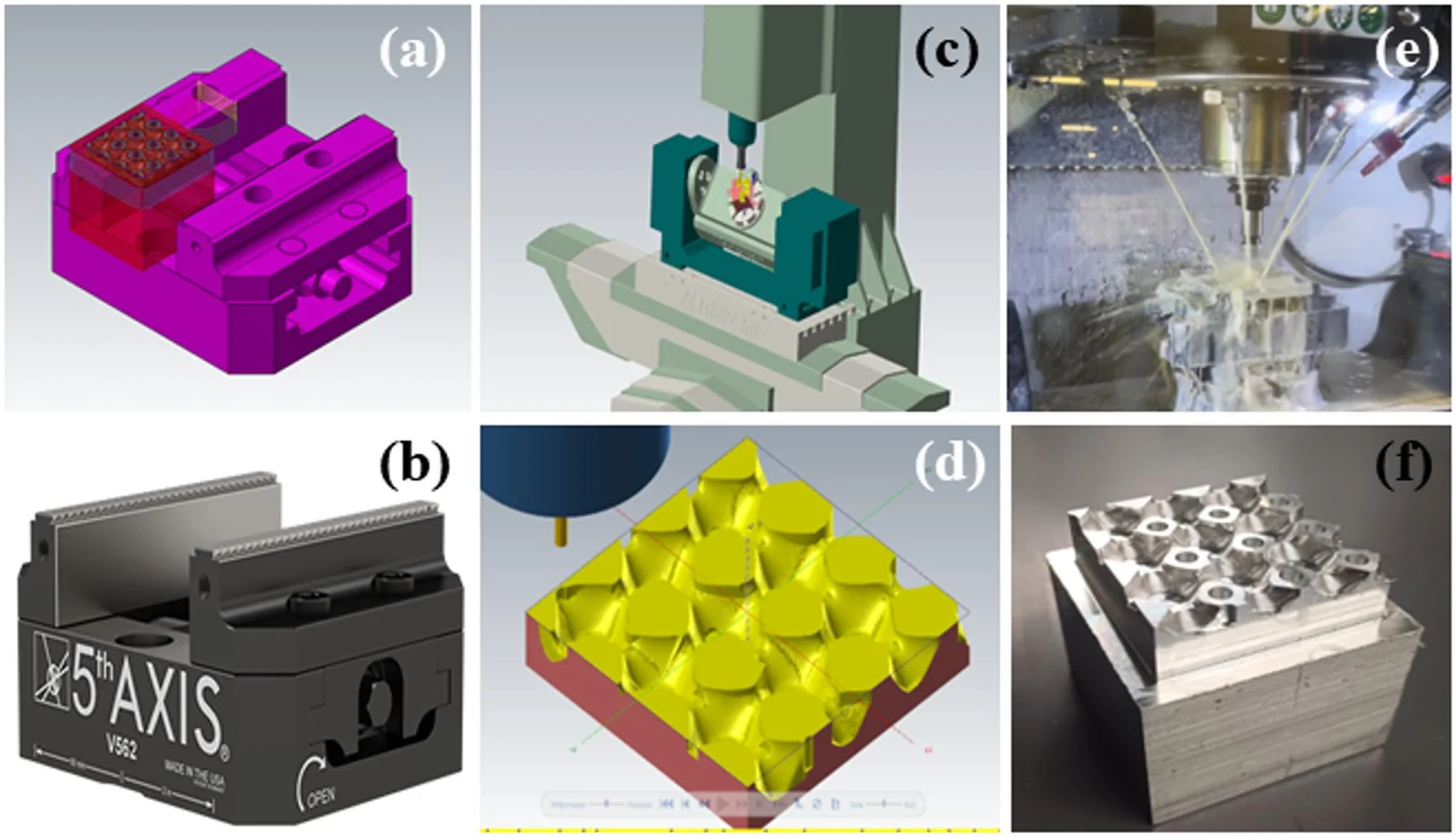
(**a**) Virtual simulation of the machining setup showing the aluminum block held in a V562 self-centering vise, (**b**) Physical V562 self-centering CNC vise used during the actual machining process, (**c**) CAM simulation setup of 5-axis CNC, (**d**) Simulated toolpath over the Schwarz TPMS surface, (**e**) Actual machining of the Schwarz TPMS single layer, (**f**) Machined part showing the completed top layer of the Schwarz geometry.

To ensure precise fabrication of the single-layer Schwarz TPMS geometry, the machining process was first simulated in Mastercam before conducting any physical trials. Both 3-axis and 5-axis toolpaths were evaluated to determine the most effective strategy for handling the geometry’s complex features. The simulation environment was configured to closely replicate the actual machining setup, including a 1.96 × 1.96 × 1.96-inch Aluminum 6061-T6 block secured in a V562 self-centering CNC vise, as shown in Fig. 5(a). The corresponding physical vise used in the lab is shown in Fig. 5(b).

The 5-axis machining strategy used for fabricating the Schwarz single layer is illustrated in Fig. 5. Figure 5(c) presents the CAM simulation setup, highlighting the dynamic positioning and rotational capabilities of the 5-axis toolpath, which allows precise access to the geometry’s complex, curved surfaces without requiring multiple part reorientations. Figure 5(d) shows the tool engagement with the surface during simulation, visualizing the cutter path over the Schwarz structure’s intricate topology.

As shown in Fig. 5(e), the machining process was carried out with continuous coolant application, which is essential for maintaining dimensional accuracy, minimizing thermal distortion, and prolonging tool life—especially when working with intricate lattice geometries like Schwarz. The coolant also helps to clear chips from the cutting area, ensuring smoother tool engagement with the workpiece. Figure 5(f) presents the machined output, revealing the top single layer of the Schwarz structure.

The Schwarz TPMS single layer was machined in multiple trials to refine parameters before final fabrication using a HAAS VF3 CNC milling center equipped with a 1/16-inch flat endmill. The patterned layers were each designed to be 3.7 mm thick, while the base layer was 3 mm, resulting in a total block height of approximately 28.9 mm—slightly less than the intended 30 mm. This minor gap was deliberately introduced to accommodate a thin layer of conductive paste or filler material during stacking and bonding, which would aid in improving thermal integration between layers.

#### Trimming and finishing

**Fig. 6:**
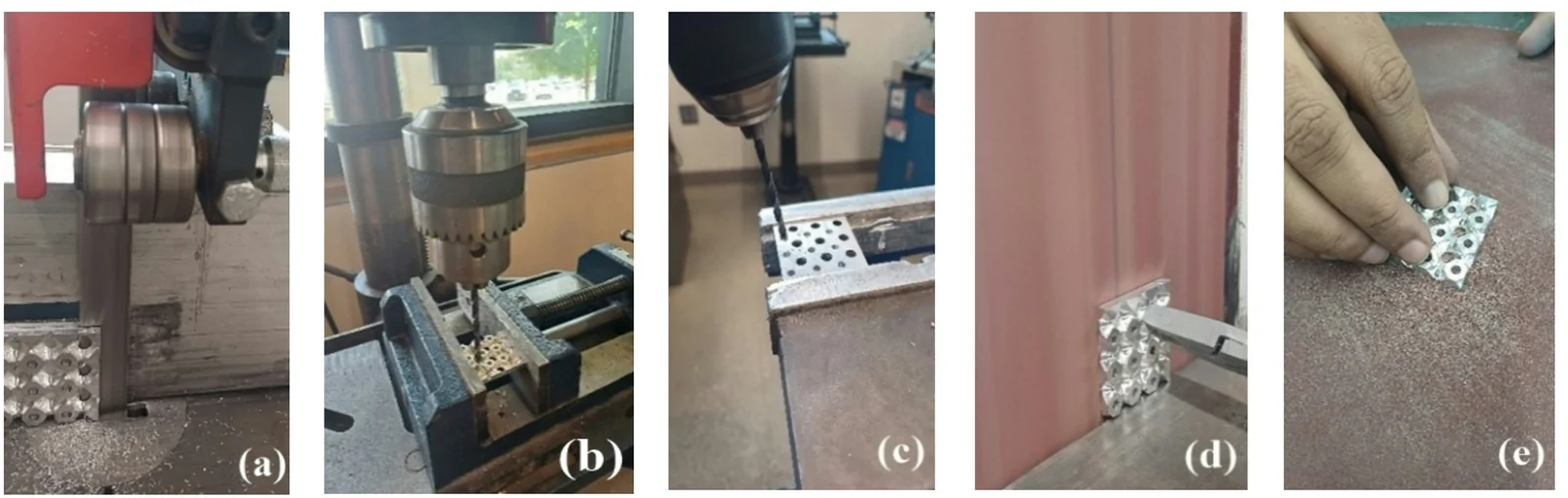
Post-processing steps for TPMS layer refinement. (**a**) Vertical band saw used to trim the edges of patterned layers; (**b**) Drill press used to align mounting holes for stacking; (**c**) Manual drilling for precise adjustments (**d**) Belt sanding for surface leveling; (**e**) Final hand sanding for thickness tuning and surface smoothness.

A trimming approach was implemented using a vertical band saw, followed by surface finishing with a belt sander and manual sanding. While this manual method produced a slightly rougher surface, it effectively achieved the desired layer thickness and preserved the structural integrity of the parts. The approach offered greater control and adaptability when working with the delicate Schwarz layers, making it a more practical solution for preparing them for stacking. This experience underscored the need to tailor post-processing techniques for complex and sensitive geometries like TPMS structures, as shown in Fig. 6.

#### Alignment and assembly

**Fig. 7:**
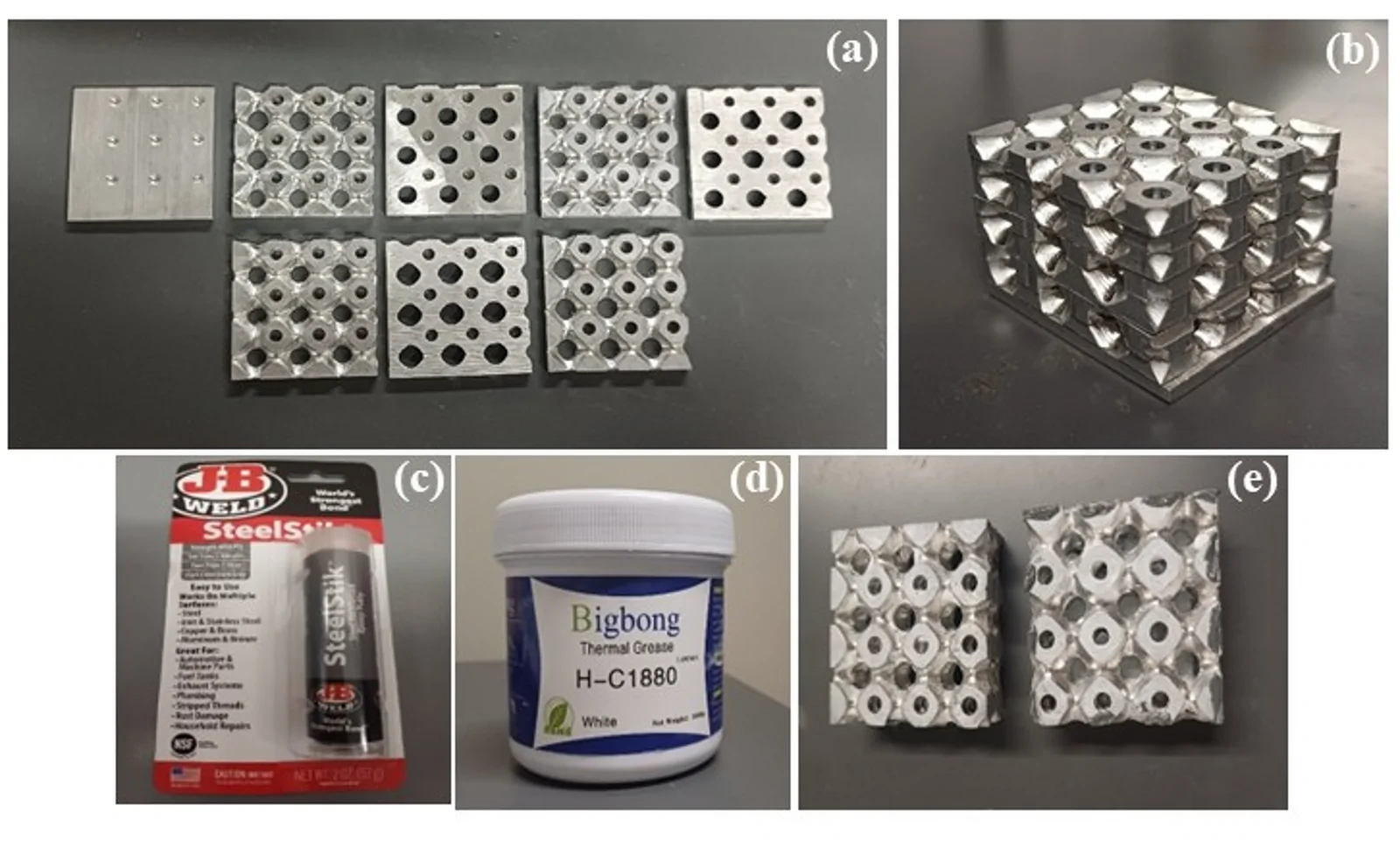
(**a**) Machined base plate and seven patterned Schwarz TPMS layers after trimming and surface finishing; (**b**) dry-fit stack of the layers manually aligned for geometric consistency before bonding; (**c**) J-B Weld SteelStik epoxy putty; (**d**) thermal conductive paste; (**e**) two separate Schwarz layers joined using epoxy and thermal paste for enhanced thermal conductivity.

Following the trimming process, seven patterned layers and one base layer were visually inspected and organized for the stacking procedure. As depicted in Fig. 7(a), the layers were laid out post-trimming and surface preparation. An initial manual alignment was carried out to verify that all holes and surfaces were properly matched. The layers were then dry-fitted to confirm alignment precision prior to the bonding step, as shown in Fig. 7(b).

A cold-welding epoxy method was implemented for bonding attempt. J-B Weld SteelStik, shown in Fig. 7(c), was selected for its effectiveness on metal surfaces and its certification for potable water applications. Prior to applying the epoxy, a thin layer of thermal conductive paste was applied between each patterned layer to improve heat transfer across the stacked assembly, as shown in Fig. 7(d). The epoxy was then kneaded and applied at the corners of each layer’s surface to provide mechanical bonding, as illustrated in Fig. 7(e). The layers were carefully aligned, pressed together, and left to cure under ambient conditions.

### Design of experiments

#### Thermoelectric cooler

**Fig. 8:**
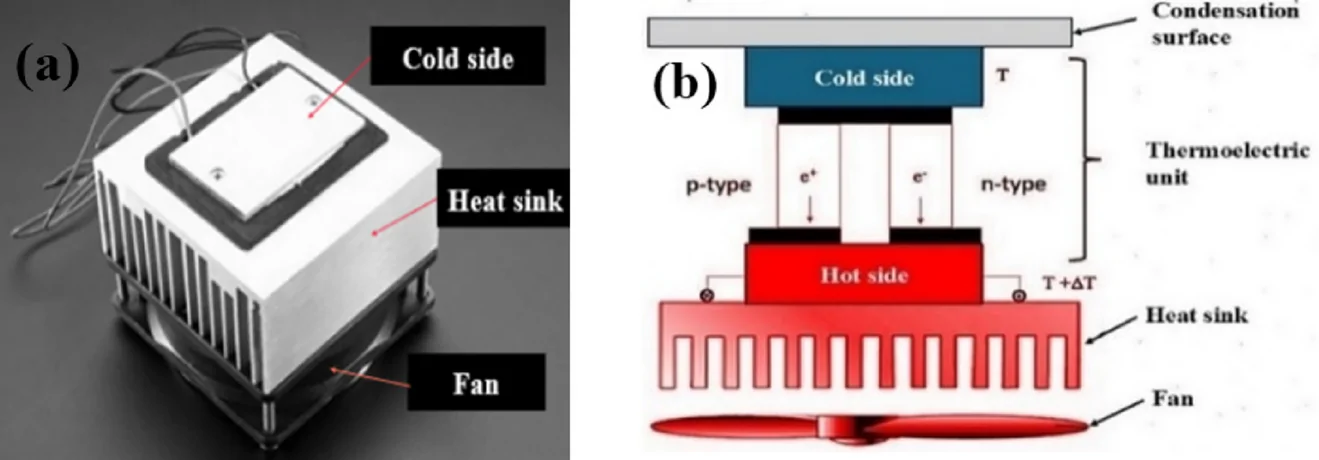
(**a**) Peltier module integrated with a heat sink and cooling fan, (**b**) Diagram illustrating the components and working principle of the Peltier system.

The experimental setup for evaluating the condensation performance of the Schwarz TPMS block is based on a TEC system utilizing a Peltier module, as shown in Fig. 8. The Peltier device operates on the principle of the Peltier effect, where direct current drives heat transfer across dissimilar semiconductor materials (p-type and n-type), creating a temperature differential. The cold side of the module, placed at the top, serves as the condensation surface where moisture from ambient air condenses. To ensure efficient heat rejection, the hot side is coupled to an aluminum heat sink and actively cooled using a fan, which helps maintain a temperature gradient across the device.

The cooling capacity of the thermoelectric module is governed by the combined effects of thermoelectric pumping, electrical resistance, and thermal conduction and can be expressed as Eq. 11.

11 $$\:{\mathrm{Q}}_{\mathrm{c}}={\upalpha\:}\mathrm{I}{\mathrm{T}}_{\mathrm{c}}-\frac{1}{2}{\mathrm{I}}^{2}\mathrm{R}-\mathrm{K}({\mathrm{T}}_{\mathrm{h}}-{\mathrm{T}}_{\mathrm{c}})$$

where $\:{\mathrm{Q}}_{\mathrm{c}}$ is the cooling power, $\:{\upalpha\:}$ is the Seebeck coefficient, $\:\mathrm{I}$ is the operating current, $\:\mathrm{R}$ is the electrical resistance, $\:\mathrm{K}$ is the thermal conductance, and $\:{\mathrm{T}}_{\mathrm{h}}$ and $\:{\mathrm{T}}_{\mathrm{c}}$ are the hot- and cold-side temperatures, respectively. The applied voltage $\:\mathrm{V}$ is related to the operating current, shown in Eq. 12, indicating that variations in voltage directly influence the current and, consequently, the cooling performance of the module.

12 $$\:\mathrm{V}=\mathrm{I}\mathrm{R}+{\upalpha\:}({\mathrm{T}}_{\mathrm{h}}-{\mathrm{T}}_{\mathrm{c}})$$

This voltage-dependent behavior provides the physical basis for the voltage-controlled experiments conducted in this study. Together, these mechanisms enable stable and continuous cooling of the upper surface, making it ideal for testing the effect of surface geometry—such as TPMS patterns—on condensation efficiency.

#### Experimental setup

**Fig. 9:**
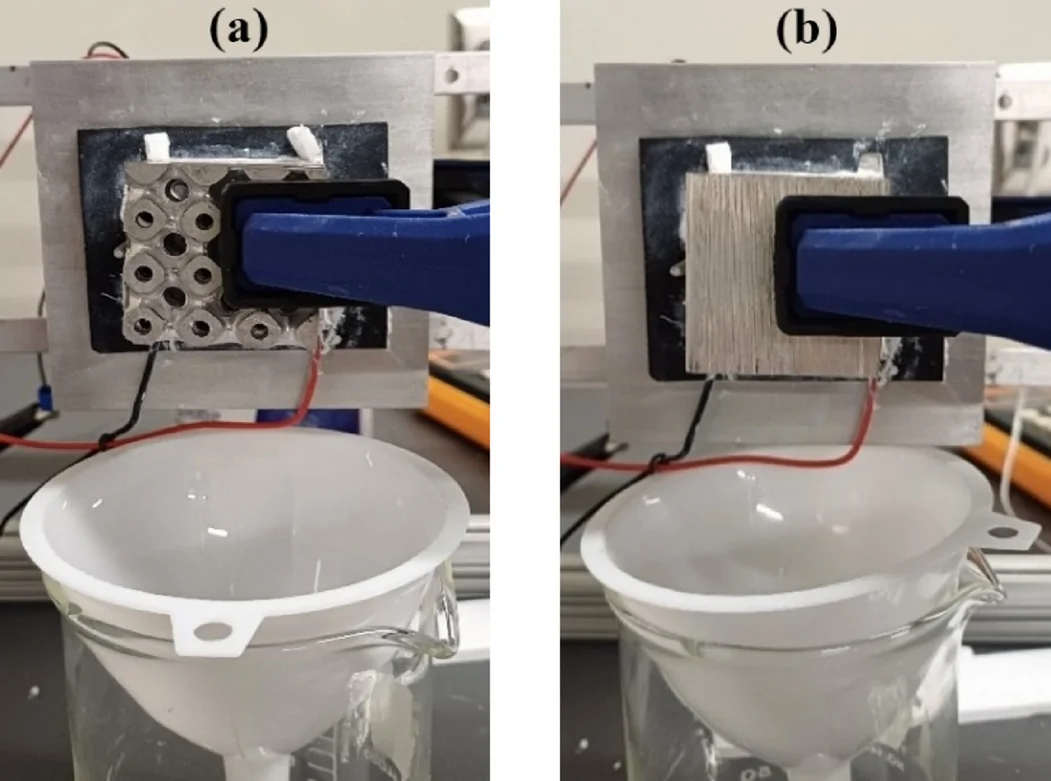
Comparative AWG experiment setup using (**a**) Schwarz TPMS structure and (**b**) solid flat aluminum block of identical external dimensions.

In this study, a comparative assessment was conducted using both a Schwarz TPMS block and a solid flat aluminum block to evaluate the performance of the Schwarz structure. Both blocks were mounted on the cold side of the Peltier device, which itself was secured within a supporting frame. To ensure proper thermal contact and stability during testing, the blocks were held in place using a clamping mechanism, as shown in Fig. 9.

#### Measurement instruments and experimental apparatus

Both the Peltier module and the cooling fan were powered using a DC power supply. Accurate temperature measurement was critical in this experiment and was performed using a digital thermometer equipped with a K-type thermoprobe, which is compatible with aluminum surfaces. Relative humidity (RH) and ambient room temperature were recorded using a digital hygrometer. To monitor environmental airflow, a handheld anemometer was used, which confirmed that air velocity was effectively zero, ensuring that no external convection influenced the condensation process.

The condensed water was collected in a beaker placed directly beneath the cold surface and measured by weight using a digital scale. Given that the density of water at room temperature is approximately 1 g/cm³, the collected mass (in grams) was directly converted to volume (in milliliters) using a 1:1 ratio. To minimize evaporation losses during the collection period, a plastic funnel was placed over the beaker to enclose the water collection zone.

#### Factors and experimental design

The experiment was organized around four key categories of variables: input factors, constant parameters, noise factors, and output responses. The primary input variables were the applied voltage to the thermoelectric module (ranging from 4 V to 7 V) and the geometry type, comparing the Schwarz TPMS block to a solid aluminum block. To maintain consistency across trials, several conditions were held constant, including fan operation, a fixed run time of one hour, and a standardized block orientation. Environmental conditions—such as relative humidity (ranging from 57 to 59%), dew point, room temperature, and current draw—were treated as noise factors; they were recorded but not controlled, to better simulate realistic operating conditions. The dew point temperature, which governs the onset of condensation, was estimated using the Magnus–Tetens approximation, shown in Eqs. 13 and 14.

13 $$\:\begin{array}{cccc}&\:{\mathrm{T}}_{\mathrm{d}\mathrm{p}}=\frac{\mathrm{b}{\hspace{0.17em}}{\upgamma\:}(\mathrm{T},\mathrm{R}\mathrm{H})}{\mathrm{a}-{\upgamma\:}(\mathrm{T},\mathrm{R}\mathrm{H})}&\:&\:\end{array}$$

14 $$\:\begin{array}{cccc}&\:{\upgamma\:}(\mathrm{T},\mathrm{R}\mathrm{H})=\mathrm{l}\mathrm{n}\left(\frac{\mathrm{R}\mathrm{H}}{100}\right)+\frac{\mathrm{a}\mathrm{T}}{\mathrm{b}+\mathrm{T}}&\:&\:\end{array}$$

where $\:{\mathrm{T}}_{\mathrm{d}\mathrm{p}}$ is the dew point temperature (°C), $\:\mathrm{T}$is the ambient air temperature (°C), and $\:\mathrm{R}\mathrm{H}$ is the relative humidity (%). The empirical constants $\:\mathrm{a}=17.27$ and $\:\mathrm{b}={237.7}^{\circ\:}\mathrm{C}$ were adopted, which are valid for typical atmospheric conditions. Condensation was expected when the cold-side surface temperature dropped below the corresponding dew point value, and this criterion was used to verify suitable operating conditions during all experiments.

The main responses measured were the volume of water collected and the temperature at the cold surface. A detailed overview of all variables is provided in Table 1, which outlines the experimental framework, and the criteria used to evaluate performance.

**Table 1:** Summary of experimental factors used in evaluating AWG performance.

Factor Type	Factors	Details / Setting Values
Input Factors	Voltage	4 V to 7 V
	Geometry Type	Schwarz TPMS / Solid block
Constant Settings	Fan	On
	Time	1 h
	Orientation	Standardized
Noise Factors	Relative Humidity	57–59% (lab)
	Dew Point	14.03–14.56 °C (lab)
	Room Temperature	~ 23 °C (lab)
	Electrical Current	Varies with applied voltage
Output Factors	Amount of Water	Measured in grams
	Cold Side Temperature	°C
Selection Criteria	Optimal Condition	Maximum water collected (in grams)

## Results

### Performance metrics for Schwarz

**Fig. 10:**
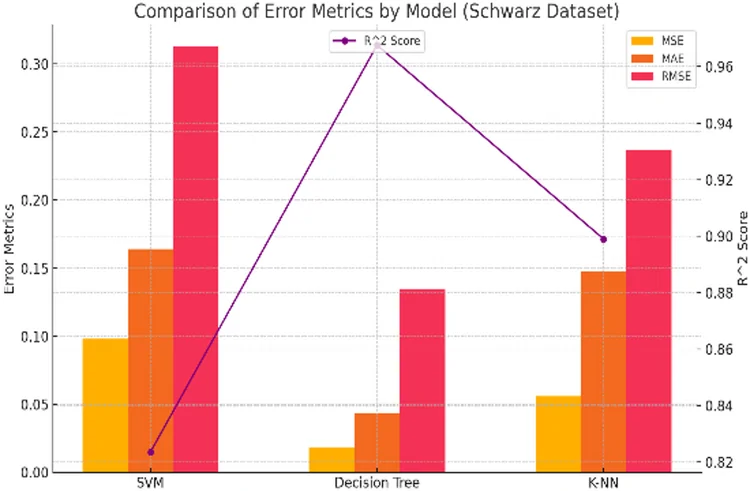
Comparison of performance metrics (MSE, MAE, RMSE) and R² scores for SVM, Decision Tree, and K-NN models applied to the Schwarz structure dataset.

The performance of the SVM, Decision Tree Regression, and KNN models in predicting the top surface temperature of Schwarz structures is evaluated using MSE, MAE, RMSE, and R². These metrics provide a comprehensive assessment of each model’s ability to capture relationships between input parameters and the target variable, as shown in Fig. 10.

The SVM model demonstrates moderate predictive performance, with an MSE of 0.0980 and RMSE of 0.3130, indicating higher error margins compared to the other models. The MAE of 0.1639 reflects a moderate average deviation, while an R² score of 0.8236 suggests it explains 82.36% of the variance in the target variable. Although SVM captures some non-linear relationships, its accuracy remains limited. The Decision Tree Regression model outperforms the other models, achieving the lowest error metrics (MSE: 0.0180, RMSE: 0.1342, MAE: 0.0433) and the highest R² score of 0.9676, explaining 96.76% of the variance. Its ability to effectively model complex non-linear interactions makes it the most reliable model for predicting top surface temperature. The KNN model performs better than SVM but is less accurate than the Decision Tree, with an MSE of 0.0561, RMSE of 0.2368, and R² of 0.8990. While KNN effectively captures localized patterns, it struggles to model intricate non-linear trends, making it less suitable than Decision Tree for this dataset.

### Relationship diagrams for schwarz

**Fig. 11:**
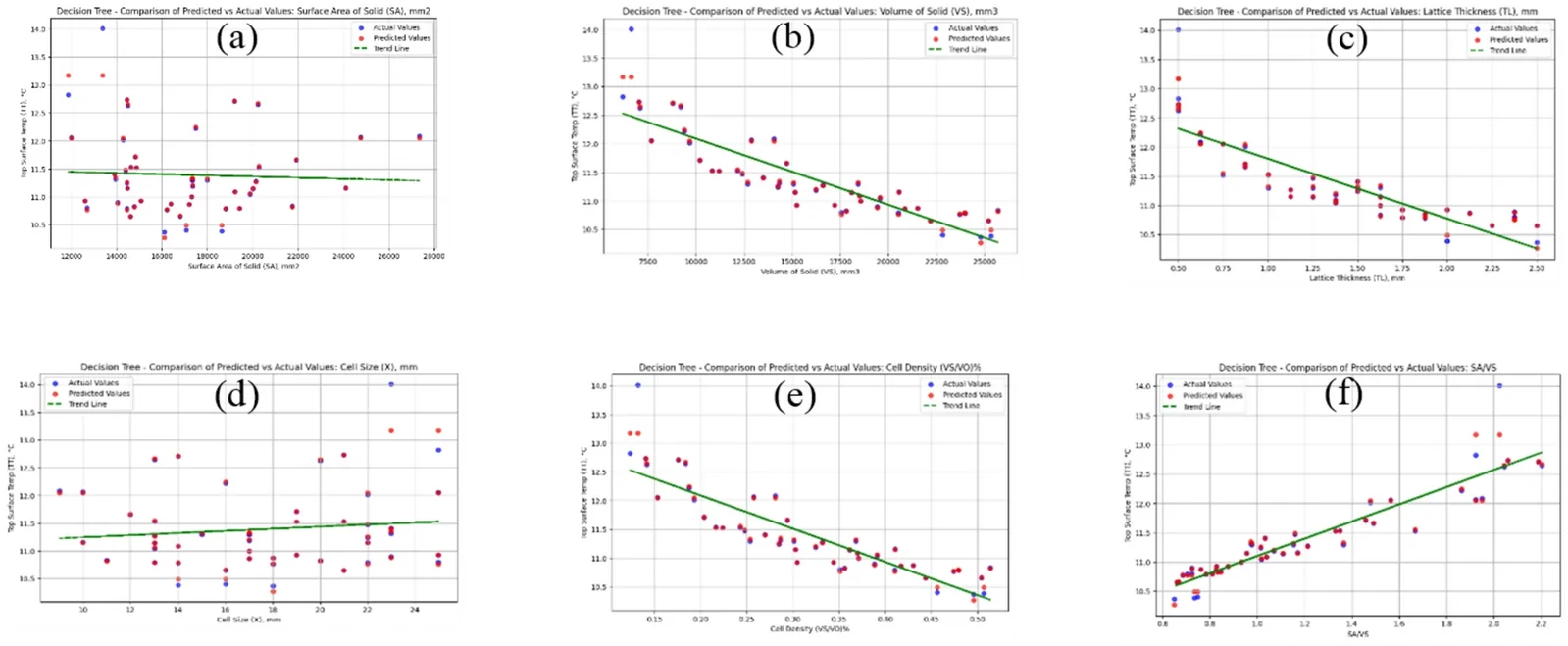
Decision tree model relationships for Schwarz structure: (**a**) Surface area of solid vs. top surface temperature, (**b**) Volume of solid vs. top surface temperature, (**c**) Lattice thickness vs. top surface temperature, (**d**) Cell size vs. top surface temperature, (**e**) Cell density vs. top surface temperature, and (**f**) Surface area-to-volume ratio (SA/V) vs. top surface temperature.

For the Schwarz structure, the relationships between surface parameters and top surface temperature exhibit both linear and non-linear trends (Fig. 11). The Decision Tree model is identified as the best fit, though SVM and KNN show similar trends.

A non-linear relationship is observed between surface area and top surface temperature, indicating complex thermal interactions. In contrast, solid volume, lattice thickness, and cell density follow a linear decreasing trend, suggesting that reducing these parameters increases the top surface temperature. Conversely, SA/V ratio exhibits a linear increasing trend, showing that higher ratios lead to higher surface temperatures.

Although the relationship diagrams in Fig. 11 reveal useful thermal trends, these trends must be interpreted alongside AWG functional requirements. As shown in Fig. 11(b) and Fig. 11(c), increasing solid volume and lattice thickness generally lowers the top surface temperature due to improved conduction through thicker material pathways. However, this improvement comes with a trade-off: when lattice thickness becomes too high (above ~ 2 mm), the geometry loses significant porosity, which reduces the exposed surface area needed for droplet nucleation and moisture capture.

For this reason, the final optimized design does not use the maximum lattice thickness. Instead, a thickness of 1.0 mm is selected because it can provide the best balance between: (i) sufficient conduction to reduce surface temperature (trend shown in Fig. 11 (c)), (ii) adequate mechanical strength for subtractive manufacturing, and (iii) high accessible surface area, which is essential for generating more droplets during AWG (related to Fig. 11(a) and Fig. 11(f)).

Similarly, while Fig. 11(e) shows that higher cell density reduces surface temperature and Fig. 11(f) shows that lower SA/V ratios correspond to lower temperature, condensation performance depends on both cooling and surface availability. The chosen parameters—cell size 7 mm and lattice thickness 1 mm—maximize usable surface area while maintaining a sufficiently low temperature to sustain continuous condensation without inducing premature frost formation.

### Completed schwarz block

This study demonstrates that Schwarz TPMS structures can be successfully fabricated using subtractive manufacturing techniques. A layer thickness of 3.7 mm was selected to accurately preserve the final optimized Schwarz TPMS geometry, characterized by a 7 mm unit cell size and a 1 mm lattice thickness, while providing an optimal balance between geometric fidelity and mechanical stability during machining.

After CNC milling, each individual Schwarz layer was initially rough-cut using a vertical band saw and subsequently refined through manual trimming and sanding to achieve the dimensional accuracy required for proper alignment and stacking. Key geometric features, including lattice thickness and unit cell dimensions, were measured at multiple locations using a digital caliper and compared with the original CAD model. The results indicated an average dimensional deviation of ± 0.07 mm, with a maximum deviation of 0.14 mm, corresponding to a relative error below 3%. Minor deviations were primarily observed near curved internal surfaces and thin lattice edges, where tool accessibility and local deflection effects were most pronounced.

Initial bonding trials using soldering techniques were unsuccessful due to uneven heat distribution and insufficient mechanical strength. As an alternative, a cold-weld epoxy was applied at the corners of each layer, along with thermal conductive paste between layers to improve heat transfer. This approach resulted in a strong, thermally functional assembly.

**Fig. 12:**
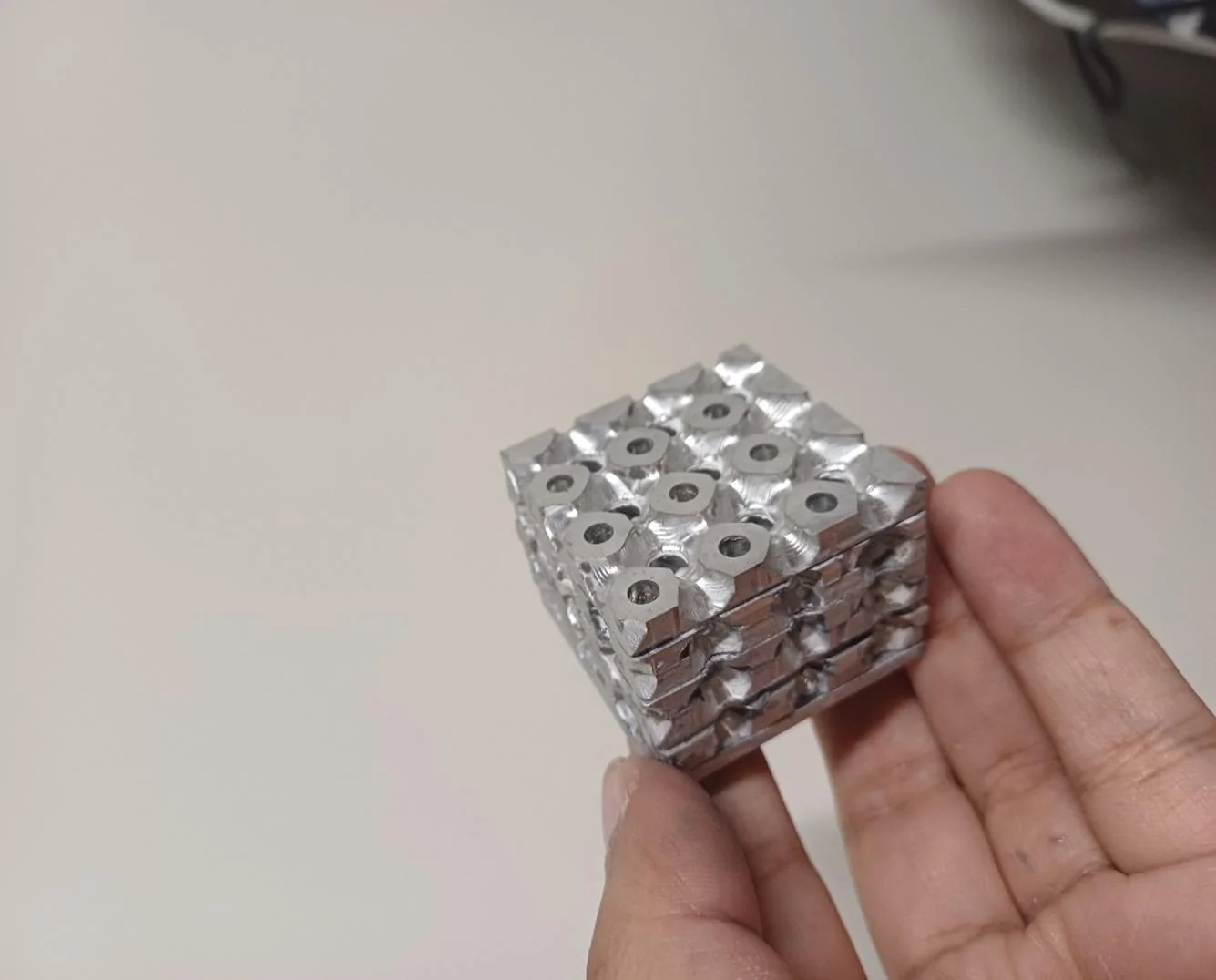
Final assembled Schwarz block after curing, showing good structural integrity and alignment.

The final stacked Schwarz block closely matched the original digital model and exhibited good structural integrity during handling and initial performance evaluation. Figure 12 shows the fully assembled block after curing, highlighting the successful alignment and uniformity of the layers. This outcome validates the feasibility of using CNC machining for fabricating TPMS geometries, which are often considered difficult to produce using conventional subtractive methods.

### Temperature profile comparison

Table 2 presents detailed temperature measurements taken from 13 fixed points on both the Schwarz TPMS and solid aluminum blocks after 1 h of operation across a voltage range of 4 V to 7 V. These readings form the foundation for generating 3D thermal distribution plots and calculating the average cold-side temperatures. The results clearly show that as the applied voltage increases, the surface temperatures decrease consistently across both block geometries.

**Table 2:** Temperature distribution across different locations on Schwarz and Solid blocks under various voltage levels.

Geometry Type	Voltage (V)	Front center	Front left	Front right	Front bottom	Front top	Top mid center	Top mid back	Left mid center	Left mid back	Right mid center	Right mid back	Bottom mid center	Bottom mid back
Schwarz block	4	9.80	12.00	9.30	9.40	9.80	8.50	6.80	8.60	8.00	9.10	7.90	8.20	6.60
	5	6.60	7.90	7.60	6.90	7.50	6.20	5.20	7.20	4.50	5.80	3.60	5.80	3.60
	6	4.60	6.90	6.00	6.50	5.00	3.80	1.80	6.00	2.90	4.20	2.80	5.40	2.10
	7	2.70	4.70	3.30	4.00	4.10	2.20	0.20	2.30	0.10	2.50	0.30	1.70	-0.10
Solid block	4	7.60	8.60	8.90	8.40	8.50	7.40	7.20	8.30	8.20	8.20	7.70	7.30	6.20
	5	5.60	6.30	6.50	6.20	5.90	5.80	5.60	6.00	5.80	5.90	5.60	5.80	5.40
	6	3.80	5.20	5.70	5.90	4.40	4.40	4.30	4.50	4.20	4.40	3.60	4.70	3.40
	7	2.20	3.20	4.40	4.00	3.20	1.80	1.30	1.90	1.20	1.90	1.30	2.10	1.80

Note: All temperature values are in degrees Celsius (°C).

The 3D temperature distribution plots were created using a Python script that applies spatial interpolation to visualize internal temperature gradients within a cubic block at varying voltage levels. The script utilizes NumPy for data processing, SciPy’s griddata function for linear interpolation across a 3D grid, and Matplotlib for generating the visualizations. Temperature data was collected from 13 predefined points on each block—such as front center, front left/right, front top/bottom, top mid, mid-back, left/right midpoints, and bottom sections. An additional synthetic point was added at the center-back of the block, assigned the minimum temperature among the 13 measured points. This assumption reflects typical TEC behavior, where the center-back is expected to be the coldest region due to the cooling configuration.

The visualizations include temperature slices along the central X, Y, and Z planes, along with directional labels (Front, Back, Top, Bottom, Left, Right) to enhance interpretability. Each plot corresponds to a different input voltage and illustrates how thermal energy is distributed across the block, highlighting the regions of maximum cooling effectiveness.

**Fig. 13:**
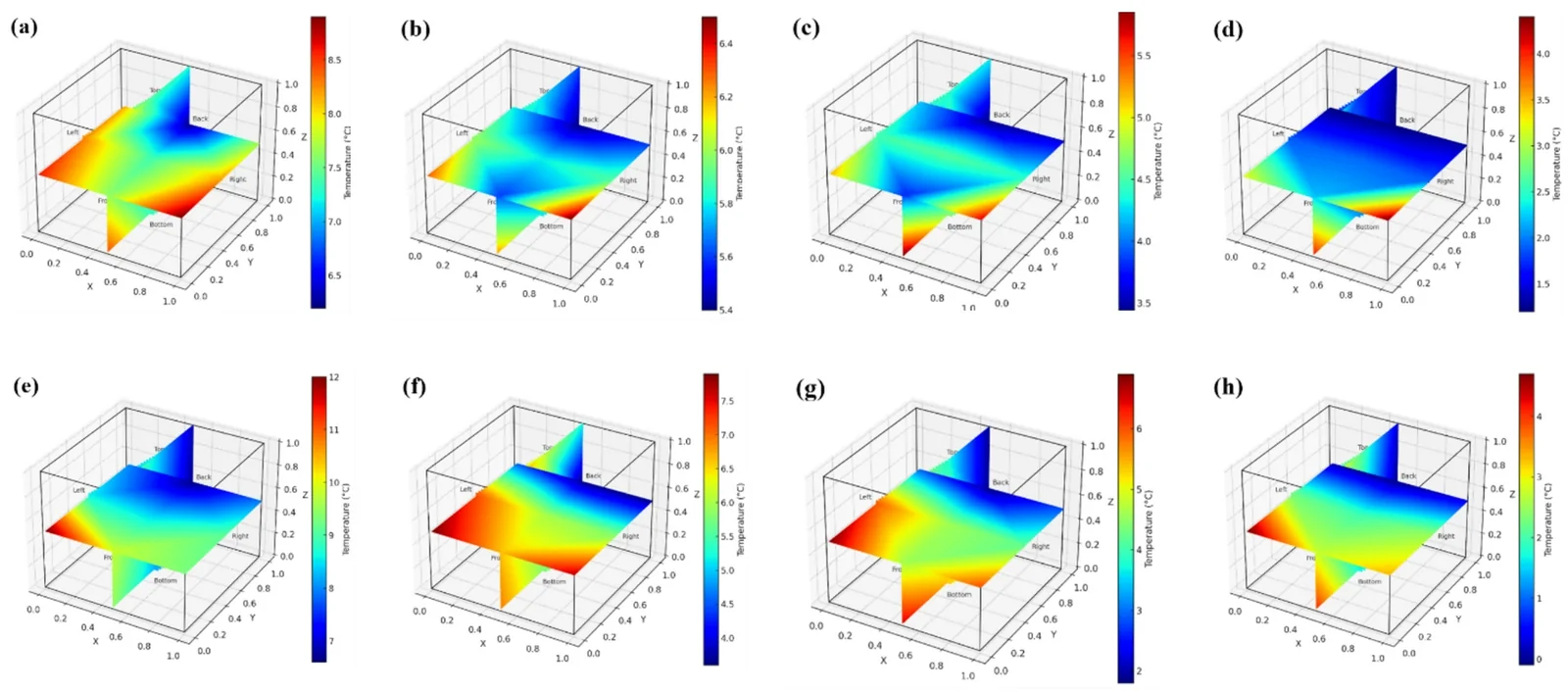
3D temperature distributions at different voltages for both geometries: (**a–d**) solid aluminum block at 4 V, 5 V, 6 V, and 7 V; (**e–h**) Schwarz TPMS block at 4 V, 5 V, 6 V, and 7 V.

Figure 13 illustrates how the internal thermal profile of the solid and Schwarz block changes with increasing voltage from 4 V to 7 V. At 4 V, the block shows the highest overall temperatures, ranging from approximately 6.5 °C to 8.6 °C, with heat concentrated in the front and central regions, resulting in a relatively mild thermal gradient, as seen in Fig. 13(a). When the voltage increases to 5 V, the overall temperature decreases, and cooler regions begin to spread more significantly throughout the block, indicating enhanced cooling performance (Fig. 13(b)). At 6 V, the temperature distribution becomes more balanced, with lower temperatures appearing across a larger volume, especially near the mid-plane (Fig. 13(c)). By 7 V, the block reaches its lowest and most uniform temperature range—approximately 1.5 °C to 4.1 °C—and exhibits a smoother gradient, reflecting maximum cooling efficiency, as depicted in Fig. 13(d).

At 4 V, shown in Fig. 13(e), the block retains relatively high temperatures, with warmer areas concentrated near the front and central regions. When the voltage is increased to 5 V, Fig. 13(f) shows a clear cooling trend, especially across the top and back surfaces, shifting the overall thermal pattern toward lower temperatures. At 6 V, depicted in Fig. 13(g), the cooling effect becomes more pronounced, with cold regions spreading more uniformly throughout the block, although some warmth remains near the front. By 7 V, as shown in Fig. 13(h), deep blue zones dominate the top and rear surfaces, indicating the most effective and uniform cooling across the entire structure.

Although both the solid and Schwarz blocks are made from aluminum, their internal temperature distributions vary significantly due to differences in structural design. The solid block displays a smoother and more uniform temperature gradient from the back to the front, which is expected given aluminum’s high thermal conductivity and the continuous nature of the material, allowing heat to distribute evenly in all directions. In contrast, the Schwarz block, consisting of stacked individual TPMS layers, exhibits a sharper temperature drop with more localized cooling near the back surface, where it interfaces with the Peltier module. This is largely due to reduced thermal continuity between layers. While thermal paste was applied between each layer to improve conduction, interfacial resistance still limits both lateral and vertical heat flow. The epoxy putty, used only at the corners for structural alignment, plays a negligible role in thermal performance.

The thermal resistance introduced by the bonding interfaces was further estimated using a one-dimensional conduction model as Eq. 15.

15 $$\:\mathrm{R}=\mathrm{t}/\left(\mathrm{k}\mathrm{A}\right)$$

where $\:\mathrm{t}$ is the interface thickness, $\:\mathrm{k}$ is the thermal conductivity, and $\:\mathrm{A}$ is the contact area. The applied thermal paste (k ≈ 5 W/m·K) and epoxy adhesive (k ≈ 0.8 W/m·K) formed interfacial layers with an average thickness of approximately 0.1 mm. Based on the effective contact area between adjacent layers, the resulting interfacial thermal resistance was estimated to be on the order of 10⁻²–10⁻¹ K/W per interface. When accumulated over multiple stacked layers, this resistance contributes to measurable temperature gradients and localized cooling effects, as observed in experimental thermal maps.

### Mean cold side temperature and water collection efficiency across different voltages

**Fig. 14:**
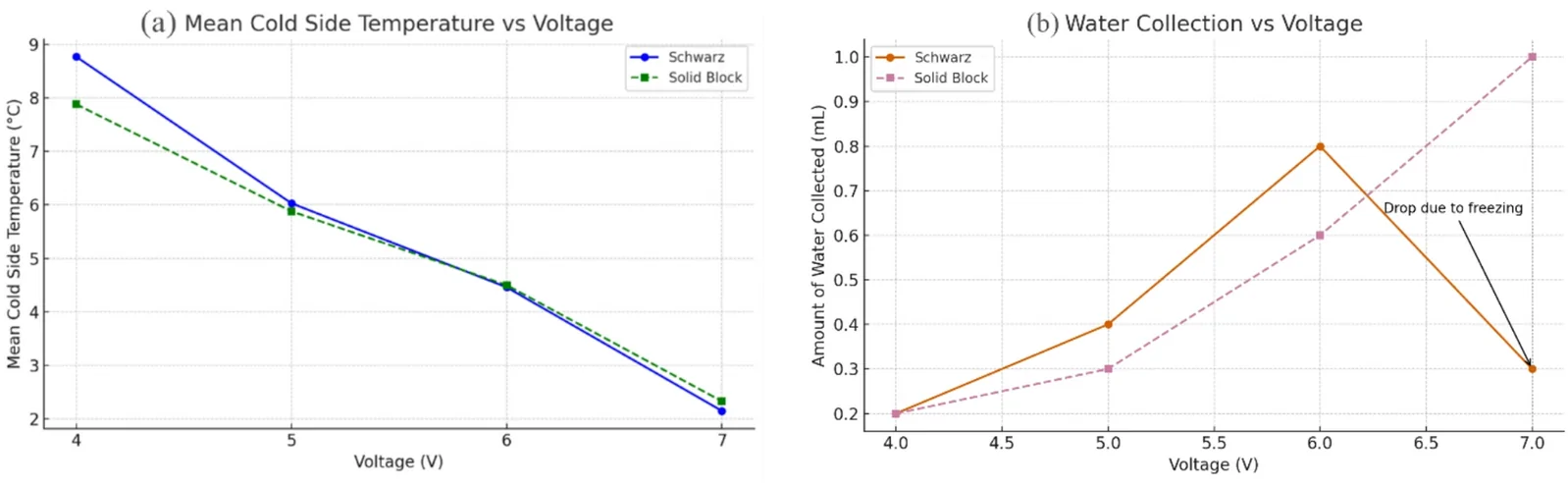
Comparison of (**a**) mean cold-side temperature and (**b**) collected water volume versus voltage for the Schwarz TPMS and solid block geometries.

As illustrated in Fig. 14(a), both the Schwarz TPMS and solid block geometries show a steady decrease in mean cold-side temperature as the applied voltage increases, reflecting the improved cooling performance of the TEC system at higher power inputs. At lower voltages (4 V and 5 V), the Schwarz structure exhibits slightly higher cold-side temperatures compared to the solid block. This may be attributed to the internal thermal resistance caused by its complex geometry, which can initially impede efficient heat transfer. However, as the voltage increases, this temperature difference gradually narrows, with both blocks showing similar performance around 6 V. At 7 V, the Schwarz structure begins to outperform the solid block, achieving a slightly lower average temperature, suggesting that its intricate design becomes thermally beneficial under higher power conditions.

The water collection results, presented in Fig. 14(b), reveal a notable difference in performance between the solid block and the Schwarz TPMS structure. At intermediate voltages of 5 V and 6 V, the Schwarz block outperformed the solid block, collecting 0.4 mL and 0.8 mL of water, respectively, compared to 0.3 mL and 0.6 mL collected by solid geometry. This improvement can be attributed to the higher surface-area-to-volume ratio of the TPMS geometry, which increases the number of available nucleation sites for droplet formation. Additionally, the curved and interconnected internal pathways of the Schwarz structure facilitate droplet coalescence and gravitational drainage, allowing newly exposed cold surfaces to sustain continuous condensation. However, at 7 V, the trend reversed: the solid block collected 1.0 mL, while the Schwarz block only collected 0.3 mL, suggesting a possible performance limit or inefficiency in the TPMS structure under high power input conditions. From a heat transfer perspective, condensation occurs when the surface temperature falls below the ambient dew point, allowing water vapor to nucleate on the cooled surface. The heat transfer associated with this condensation process can be expressed as Eq. 16.

16 $$\:\mathrm{q}=\mathrm{h}\mathrm{A}({\mathrm{T}}_{\mathrm{d}\mathrm{e}\mathrm{w}}-{\mathrm{T}}_{\mathrm{s}})$$

where $\:\mathrm{q}$ is the heat transfer rate during condensation, $\:\mathrm{h}$ is the condensation heat transfer coefficient, $\:\mathrm{A}$ is the effective condensation surface area, $\:{\mathrm{T}}_{\mathrm{d}\mathrm{e}\mathrm{w}}$ is the dew point temperature, and $\:{\mathrm{T}}_{\mathrm{s}}$ is the surface temperature. The larger effective surface area provided by the TPMS geometry increases the available condensation area, thereby enhancing the overall heat transfer associated with vapor condensation. The resulting condensation rate can be related to the heat transfer rate through Eq. 17.

17 $$\:\dot{\mathrm{m}}=\frac{\mathrm{q}}{{\mathrm{h}}_{\mathrm{f}\mathrm{g}}}$$

where $\:\dot{\mathrm{m}}$ is the condensation mass rate and $\:{\mathrm{h}}_{\mathrm{f}\mathrm{g}}$ is the latent heat of vaporization of water. This relationship indicates that increased heat transfer during condensation directly contributes to higher water collection rates.

The relative improvement in water collection achieved by the Schwarz TPMS structure compared to the solid block was quantified using Eq. 18.

18 $$\:\mathrm{\%}\mathrm{\:Improvement}=\frac{{\mathrm{W}}_{\mathrm{T}\mathrm{P}\mathrm{M}\mathrm{S}}-{\mathrm{W}}_{\mathrm{s}\mathrm{o}\mathrm{l}\mathrm{i}\mathrm{d}}}{{\mathrm{W}}_{\mathrm{s}\mathrm{o}\mathrm{l}\mathrm{i}\mathrm{d}}}\times\:100$$

where $\:{\mathrm{W}}_{\mathrm{T}\mathrm{P}\mathrm{M}\mathrm{S}}$ and $\:{\mathrm{W}}_{\mathrm{s}\mathrm{o}\mathrm{l}\mathrm{i}\mathrm{d}}$ represent the collected water volumes for the TPMS and solid geometries, respectively. At intermediate operating voltages of 5–6 V, this metric indicated an enhancement of approximately 33%, confirming the effectiveness of the optimized TPMS surface in promoting condensation through increased surface area, improved droplet nucleation, and enhanced drainage pathways.

The observed decline in performance at 7 V was linked to early frost formation on the Schwarz block. The TPMS structure required less power to reach low temperatures or, at the same voltage, reached colder temperatures faster than the solid block. However, this rapid cooling triggered early icing, which blocked further droplet accumulation and disrupted continuous condensation. In contrast, the solid block’s compact and enclosed geometry retained thermal inertia more effectively, delaying frost onset and sustaining uninterrupted condensation—thereby enabling greater water collection at higher voltages.

## Discussion

### Refining predictive modeling and scaling fabrication

To further strengthen the outcomes of this work, future efforts should focus on both improving the machine learning framework and expanding fabrication trials. While this study successfully demonstrated the predictive ability of supervised learning models and validated a single CNC-fabricated Schwarz TPMS block, additional trials with varied geometric configurations are needed to fully capture the practical variability of subtractive manufacturing. Fabricating multiple samples will allow for better assessment of repeatability, robustness, and performance consistency under experimental conditions. On the modeling side, predictive accuracy can be enhanced through hyperparameter tuning of existing algorithms, expanding the dataset with additional simulation and experimental results, and exploring advanced approaches such as Random Forest, Gradient Boosting, or deep learning architectures to better capture complex non-linear relationships. Hybrid ensemble techniques could also be employed to combine the strengths of multiple models. Together, these improvements—broader experimental validation and refined predictive modeling—will provide a more comprehensive understanding of TPMS thermal behavior and accelerate the development of optimized AWG systems.

### ML-guided design optimization and parameter selection

In addition to qualitative trade-offs, the final parameter selection was quantitatively supported by the supervised learning predictions. The supervised learning model was used to evaluate all valid geometric configurations within the simulated design space and to rank them according to their predicted top surface temperature. This analysis indicated that the configuration with a lattice thickness of 1.0 mm and a unit cell size of 7 mm consistently belonged to the highest-performing group, exhibiting among the lowest predicted surface temperatures while satisfying manufacturability constraints. Alternative configurations with thicker lattices or larger cell sizes showed marginal thermal improvements but suffered from reduced porosity or machining limitations. Conversely, thinner or more open structures led to higher predicted temperatures and lower structural stability. Therefore, the selected parameter set represents a globally optimal compromise within the explored design space, as determined by the supervised learning model, and was adopted for subsequent fabrication.

### Layer thickness optimization in fabrication

**Fig. 15:**
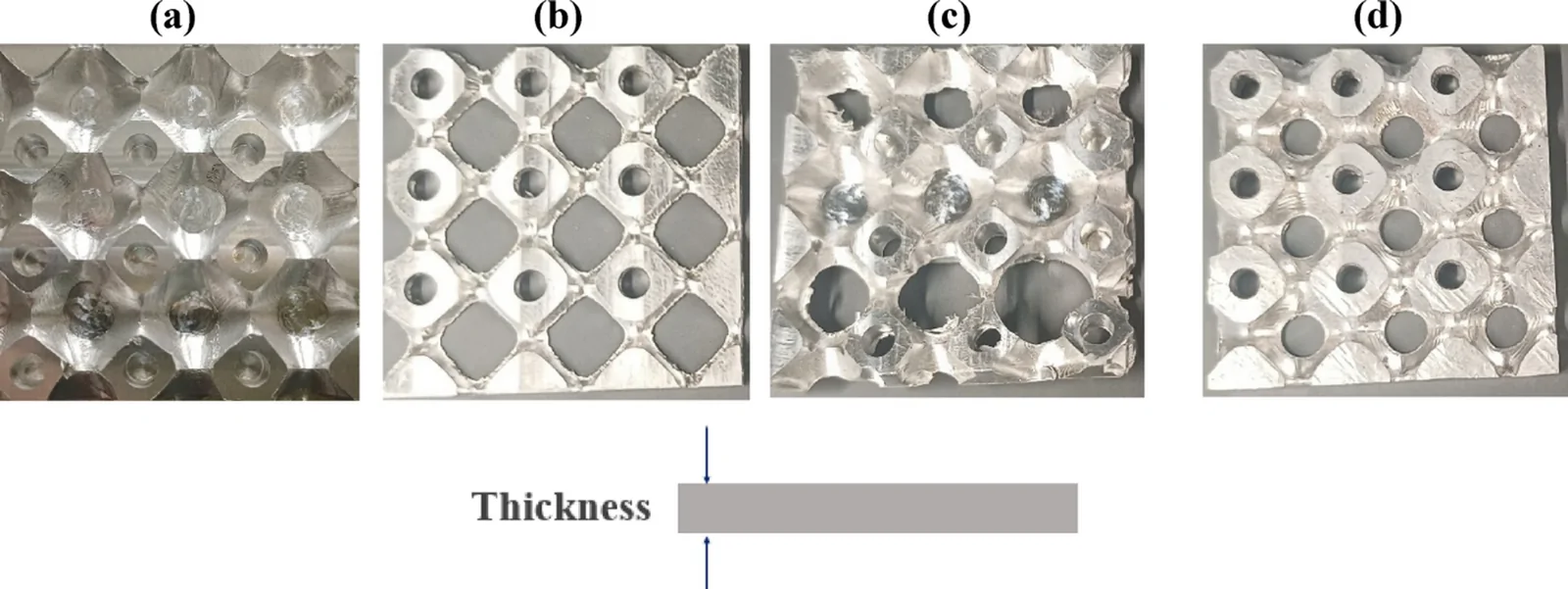
Machining outcomes for Schwarz TPMS layers (**a**) Too thick (4.5 mm), (**b**) Too thin (3 mm), (**c**) Bent due to instability, (**d**) Desired thickness (3.7 mm) with optimal precision and strength.

In the initial machining trials, several layer thicknesses were evaluated to determine the most suitable dimension for fabricating individual TPMS layers. As seen in Fig. 15(a), the 4.5 mm thick layer left behind excess material, which partially blocked the open-cell structures and reduced the geometric accuracy of the part. In contrast, Fig. 15(b) shows that the 3 mm thick layer resulted in overly thin lattice walls and larger-than-intended through-holes, compromising structural integrity. Additionally, poor rigidity during fixturing led to part deformation—such as bending and warping—during machining, as evident in Fig. 15(c).

Ultimately, a layer thickness of 3.7 mm, as shown in Fig. 15(d), was identified as the optimal balance between geometric accuracy and structural stability. This thickness enabled precise replication of the Schwarz lattice features while maintaining enough rigidity for secure clamping and reliable handling during machining.

### Trimming by CNC milling

**Fig. 16:**
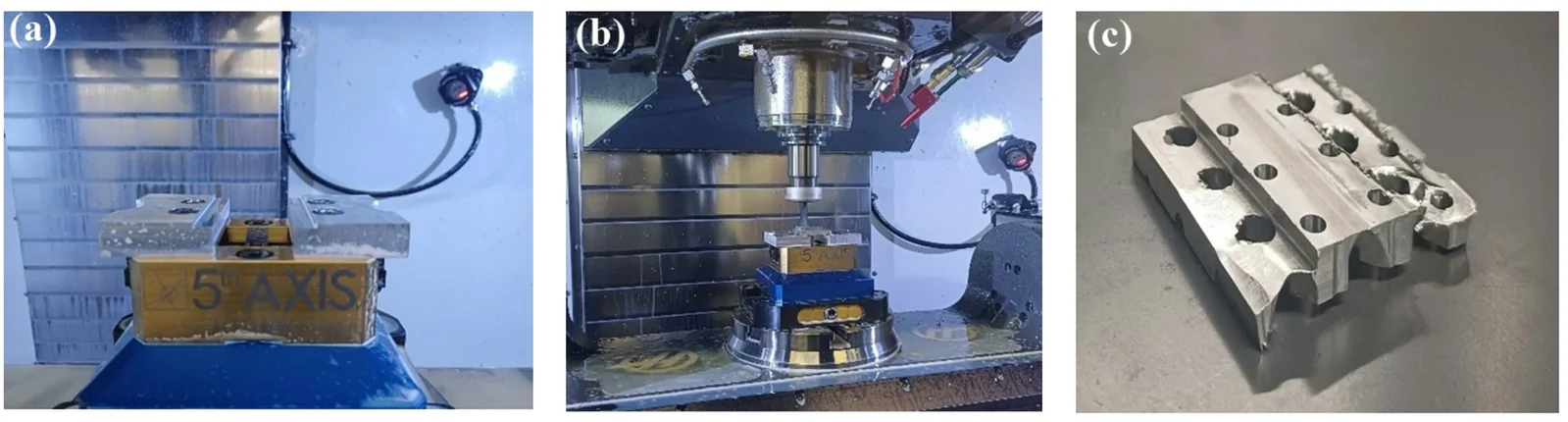
Trimming attempt using CNC milling machine. (**a**) Machining setup showing the cutting tool in position. (**b**) Customized aluminum jaws mounted on a 5-axis vise for securing thin TPMS layers. (**c**) Resulting deformation and surface damage caused by the fragility of the 3.7 mm patterned layer during the trimming process.

The first trimming attempt was conducted using the same CNC milling machine, as shown in Fig. 16(a), utilizing custom-fabricated aluminum jaws to secure the thin, patterned layers (Fig. 16(b)). However, this method proved unsuccessful. The 3.7 mm thick layers, with their numerous through-holes and limited rigidity, were prone to bending under clamping pressure, as seen in Fig. 16(c). Reducing the clamping force to prevent deformation compromised workpiece stability, leading to shifting and misalignment during machining. As a result, this CNC-based approach did not achieve the required level of accuracy or repeatability for effective trimming.

### Stacking by soldering

The first attempt to bond the layers involved soldering. A soldering iron was used along with a 60% tin, 40% lead alloy and an aluminum-compatible flux to enhance adhesion. However, this method proved ineffective. The heat distribution was uneven, and the resulting joints lacked the mechanical strength needed to keep the layers securely bonded under stress or during subsequent testing.

### Observations on freezing conditions at 7 V

**Fig. 17:**
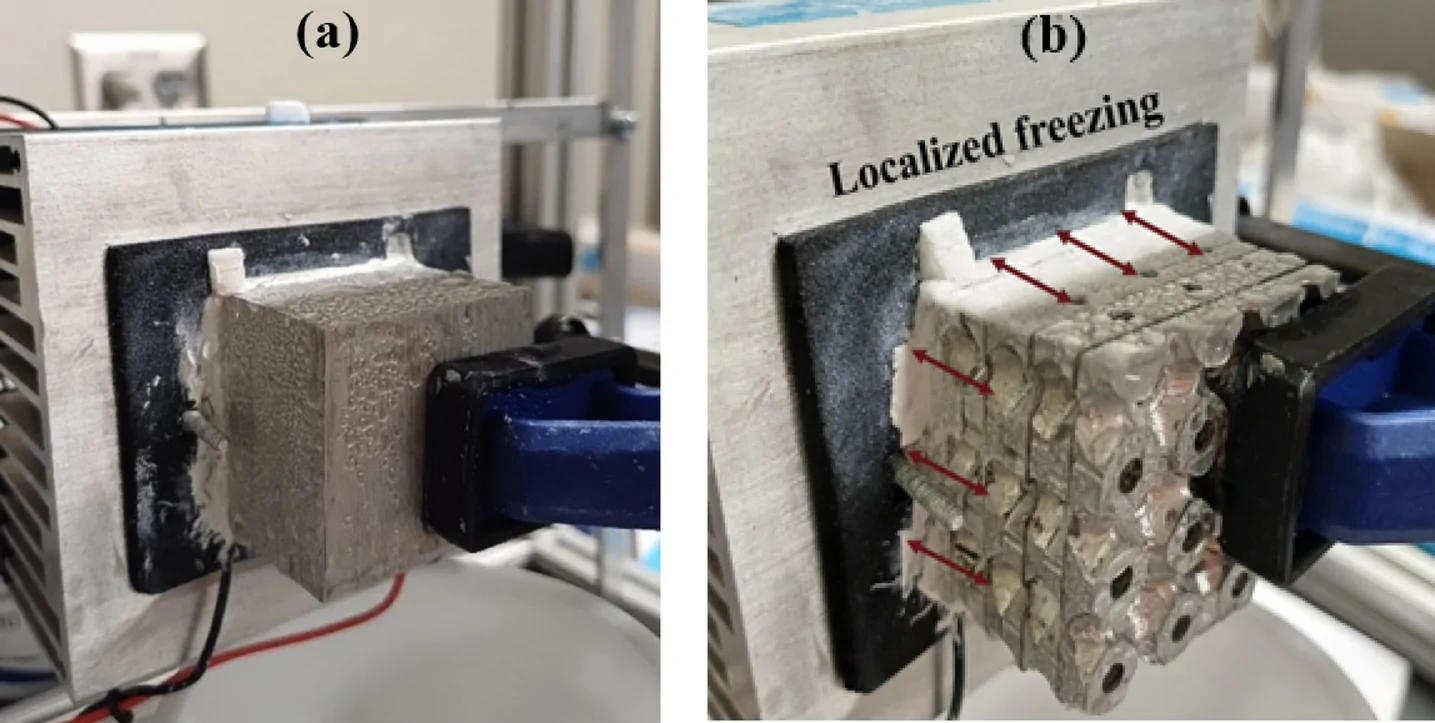
(**a**) Solid block showing uniform condensation across the surface at 7 V. (**b**) Schwarz TPMS block exhibiting localized freezing near the edges and back.

Visual inspection highlighted the contrasting freezing behaviors of the solid and Schwarz TPMS blocks. At 7 V, the solid block demonstrated uniform water condensation across its surface, as shown in Fig. 17(a), indicating efficient and consistent cooling. In comparison, the Schwarz block displayed irregular frost formation, as seen in Fig. 17(b), with ice primarily accumulating near the back side and edges. This uneven icing pattern suggests localized overcooling, likely caused by the block’s open-cell structure, which allows greater exposure to ambient air and disrupts uniform heat distribution.

### Observations on freezing conditions at 8 V

At 8 V, both the solid and Schwarz blocks eventually developed full frost coverage; however, freezing occurred earlier on the patterned Schwarz geometry. Its open and interconnected structure, while beneficial for heat transfer at moderate voltages, led to rapid surface cooling, triggering frost formation before significant condensation could take place. This premature icing effectively sealed the condensation surfaces, thereby reducing total water yield. In contrast, the solid block’s denser configuration resulted in a slower cooling rate, which delayed frost formation and allowed more time for water to condense.

### Scalability considerations for TPMS-based AWG systems

While the present study focuses on a laboratory-scale prototype using a single TEC module integrated with a TPMS condenser surface, the proposed concept can be extended to larger AWG systems through a modular design approach. In practical implementations, multiple TPMS-based condensation units may be arranged in parallel arrays, where each module consists of a TEC device, a TPMS condensation surface, and a corresponding heat rejection system. Such a modular architecture allows the overall water production capacity to scale approximately proportionally with the number of modules while maintaining manageable thermal loads on individual TEC elements.

The compact geometry and high surface-area-to-volume ratio of TPMS structures make them particularly suitable for scalable condenser configurations. By distributing condensation surfaces across multiple layers or panels, a large effective condensation area can be achieved within a relatively small device footprint. This arrangement can enhance air–surface interaction while maintaining efficient thermal conduction between the TEC cold side and the exposed condensation surface. In addition, the interconnected porous structure of TPMS geometries promotes more uniform temperature distribution and improved droplet transport pathways, which are beneficial for maintaining stable condensation behavior in larger systems.

Previous machining investigations have shown that certain TPMS geometries, particularly the Schwarz structure, can be fabricated with acceptable geometric fidelity using multi-axis CNC machining, whereas more complex geometries may present significant tool accessibility challenges. As production volume increases, batch machining significantly reduces per-unit manufacturing cost, making TPMS-based condenser components economically attractive for medium- to large-scale AWG deployment.

Despite these promising aspects, the scalability of TPMS-enhanced AWG systems will ultimately depend on system-level factors beyond geometric design alone. Parameters such as airflow management, heat rejection efficiency, environmental operating conditions, and electrical energy consumption of thermoelectric modules play critical roles in determining the overall performance of practical AWG devices. Therefore, evaluating system-level performance metrics—particularly water production relative to electrical energy consumption (e.g., liters per kilowatt-hour, L/kWh)—will be essential for assessing the real-world feasibility of large-scale TPMS-based condensation systems.

Future research should further investigate both the scalability and optimization of TPMS-based AWG systems from computational, manufacturing, and experimental perspectives. Improving the robustness and generalization of the machine learning framework will require systematic hyperparameter tuning, expansion of the training dataset, and the exploration of advanced algorithms such as Random Forest, Gradient Boosting, and deep neural networks. Although the present study employs an 80/20 train–test split to evaluate predictive performance, future studies may incorporate k-fold cross-validation and larger datasets to more rigorously assess model generalization and statistical robustness. From an experimental standpoint, multiple fabrication and testing trials with varied geometric configurations will be necessary to evaluate repeatability, quantify experimental uncertainty, and validate long-term performance stability. In addition, further investigations should consider surface engineering strategies—such as hydrophobic coatings, electrothermal defrosting methods, and hybrid additive–subtractive manufacturing approaches—to mitigate frost formation, reduce interfacial thermal resistance, and improve thermal uniformity. Collectively, these efforts will support the development of scalable, energy-efficient, and economically viable AWG systems based on geometry-driven condenser design.

## Conclusions

This study presented a novel strategy for enhancing AWG by integrating supervised machine learning with subtractively manufactured Schwarz TPMS geometries. Structural and thermal simulations were used to generate datasets, which trained supervised regression models (Decision Tree, SVM, KNN) to predict top surface temperature based on geometric parameters. Among these, the Decision Tree model demonstrated the best predictive accuracy, revealing both linear and non-linear dependencies. Guided by these insights, a Schwarz TPMS block was fabricated using multi-axis CNC machining through a layer-wise milling and stacking method, demonstrating the viability of subtractive manufacturing for complex porous structures. Experimental testing showed that the Schwarz block achieved superior water yield compared to a flat reference block at intermediate voltages (5–6 V), owing to its higher surface-area-to-volume ratio and droplet-shedding characteristics, though premature frost formation at higher voltages limited performance. Overall, the results suggest that combining machine learning–guided design with TPMS-based geometries provides a promising pathway for exploring geometry-driven optimization of condensation surfaces in TEC-based AWG systems.

## Data Availability

The datasets generated and analyzed during the current study are publicly available in a Zenodo repository: [https://doi.org/10.5281/zenodo.19212706]. Experimental materials and fabricated samples are described in sufficient detail within the manuscript to allow replication.

## Abbreviations

AWG: Atmospheric water generation
CD: Cell density
CNC: Computer numerical control
CS: Cell size
KNN: K-nearest neighbors
MSE: Mean squared error
R²: Coefficient of determination
RH: Relative humidity
RMSE: Root mean squared error
SA/V: Surface-area-to-volume ratio
SM: Subtractive manufacturing
SVM: Support vector machine
TEC: Thermoelectric cooler
TL: Lattice thickness
TPMS: Triply periodic minimal surface

## Code availability

The machine learning models and data processing scripts used in this study are publicly available in a Zenodo repository: https://doi.org/10.5281/zenodo.19212706. The repository includes all necessary files to reproduce the results presented in this work.
